# Sse1, Hsp110 chaperone of yeast, controls the cellular fate during endoplasmic reticulum stress

**DOI:** 10.1093/g3journal/jkae075

**Published:** 2024-04-05

**Authors:** Mainak Pratim Jha, Vignesh Kumar, Asmita Ghosh, Koyeli Mapa

**Affiliations:** Protein Homeostasis Laboratory, Department of Life Sciences, School of Natural Sciences, Shiv Nadar Institution of Eminence, Delhi-NCR, Gautam Buddha Nagar, Greater Noida, Uttar Pradesh 201314, India; Chemical and Systems Biology Unit, CSIR–Institute of Genomics and Integrative Biology, New Delhi 110025, India; Academy of Scientific and Innovative Research (AcSIR), Ghaziabad 201002, Uttar Pradesh, India; Chemical and Systems Biology Unit, CSIR–Institute of Genomics and Integrative Biology, New Delhi 110025, India; Academy of Scientific and Innovative Research (AcSIR), Ghaziabad 201002, Uttar Pradesh, India; Protein Homeostasis Laboratory, Department of Life Sciences, School of Natural Sciences, Shiv Nadar Institution of Eminence, Delhi-NCR, Gautam Buddha Nagar, Greater Noida, Uttar Pradesh 201314, India

**Keywords:** molecular chaperone, protein homeostasis, heat shock protein 110, endoplasmic reticulum unfolded protein response, endoplasmic reticulum stress

## Abstract

Sse1 is a cytosolic Hsp110 molecular chaperone of yeast, *Saccharomyces cerevisiae*. Its multifaceted roles in cellular protein homeostasis as a nucleotide exchange factor (NEF), as a protein-disaggregase and as a chaperone linked to protein synthesis (CLIPS) are well documented. In the current study, we show that *SSE1* genetically interacts with *IRE1* and *HAC1*, the endoplasmic reticulum-unfolded protein response (ER-UPR) sensors implicating its role in ER protein homeostasis. Interestingly, the absence of this chaperone imparts unusual resistance to tunicamycin-induced ER stress which depends on the intact Ire1-Hac1 mediated ER-UPR signaling. Furthermore, cells lacking *SSE1* show inefficient ER-stress-responsive reorganization of translating ribosomes from polysomes to monosomes that drive uninterrupted protein translation during tunicamycin stress. In consequence, the *sse1*Δ strain shows prominently faster reversal from ER-UPR activated state indicating quicker restoration of homeostasis, in comparison to the wild-type (WT) cells. Importantly, Sse1 plays a critical role in controlling the ER-stress-mediated cell division arrest, which is escaped in *sse1*Δ strain during chronic tunicamycin stress. Accordingly, *sse1*Δ strain shows significantly higher cell viability in comparison to WT yeast imparting the stark fitness following short-term as well as long-term tunicamycin stress. These data, all together, suggest that cytosolic chaperone Sse1 is an important modulator of ER stress response in yeast and it controls stress-induced cell division arrest and cell death during overwhelming ER stress induced by tunicamycin.

## Introduction

Sse1 is a member of the Hsp110 group of molecular chaperones found in yeast, *Saccharomyces cerevisiae*. To date, it is well established that Hsp110s act as nucleotide exchange factors (NEF) for its Hsp70 partners (Dragovic *et al*. 2006; Polier *et al*. 2008; Andreasson *et al*. 2008a; Andreasson *et al*. 2008b). Hsp110s are prominently similar to Hsp70 chaperones in their domain organization and structure (Liu and Hendrickson 2007; Polier *et al*. 2008; Schuermann *et al*. 2008). Like Hsp70s, Hsp110s are also two-domain proteins, with a ∼45 kDa N-terminal nucleotide binding domain and a ∼25 kDa peptide or substrate binding domain (PBD or SBD) at the C-terminus. The structural and molecular basis of the NEF function of yeast Hsp110, Sse1, has been explored in great detail in the last decade (Polier *et al*. 2008; Schuermann *et al*. 2008; Andreasson *et al*. 2008a). Although there is a plethora of information regarding the domain allostery of Hsp70s, the same is still elusive for Sse1 and other Hsp110s. Previously, we showed that the ATP-hydrolysis-driven conventional domain movements found in Hsp70s are absent in Sse1, although there are distinctive noncanonical domain movements found in this protein upon ATP hydrolysis (Kumar *et al*. 2020). Sse1 is also distinctly different from its Hsp70 partners in its cellular functions. Apart from its well-known function as a NEF for Hsp70 partner proteins like Ssa1 or Ssb1 (Shaner *et al*. 2005; Dragovic *et al*. 2006), Sse1 is also known to act as a cochaperone for Hsp90 (Liu *et al*. 1999) or assists in protein disaggregation in association with Hsp70 and Hsp104 chaperones (Kumar *et al*. 2020). Due to its co-regulated expression pattern with cytosolic translation machinery, Sse1 also belongs to chaperones Linked to Protein Synthesis (CLIPS) (Albanese *et al*. 2006). CLIPS are a group of cytosolic chaperones that are transcriptionally downregulated during stress (under different cellular stresses like heat shock, osmotic shock, oxidative stress, etc.) and are transcriptionally co-regulated with protein translation apparatus. They are distinct from the stress-induced chaperones designated commonly as heat shock proteins or HSPs. Among CLIPS, only Ssa1 and Sse1 were found to be overexpressed during heat stress but were transcriptionally repressed during most of the other stresses (Albanese *et al*. 2006). Very recently, another function of Sse1 in endoplasmic reticulum (ER) protein homeostasis was revealed. In yeast, ER protein homeostasis is maintained by Ire1-Hac1 mediated ER-unfolded protein response (ER-UPR) (Wu *et al*. 2014; Higuchi-Sanabria *et al*. 2018; Hetz *et al*. 2020). This is the most conserved signaling pathway of ER-UPR across all eukaryotes where an ER transmembrane protein, Ire1 senses the stress within ER lumen through its luminal domain. Upon sensing the stress, Ire1 is activated leading to its oligomerization and trans-autophosphorylation by its cytosolic kinase domain. The activated Ire1 next removes an intron from the *HAC1* mRNA by a noncanonical splicing event by its RNAse activity (Wu *et al*. 2014; Higuchi-Sanabria *et al*. 2018; Hetz *et al*. 2020). The spliced *HAC1* mRNA is translated to Hac1 protein (Hac1p) which acts as a transcription factor to upregulate the ER-UPR and ER-associated degradation of proteins (ERAD) genes. Earlier, it was shown that some of the ERAD substrates are stabilized by Sse1 indicating that the chaperone might be important in ER proteostasis (Hrizo *et al*. 2007). Very recently, it was shown that cells deleted of *SSE1* are less efficient in ER-reflux of proteins, a newly described quality control pathway parallel to ERAD which is important for upkeep of the ER protein homeostasis (Igbaria *et al*. 2019). Thus, it is evident that Sse1 has a cross-compartment role in maintaining protein homeostasis in yeast.

In this work, in an attempt to understand the role of Sse1 during ER stress, we subjected the *SSE1* deletion strain to ER stressor tunicamycin abbreviated hereafter as Tm. Tm inhibits N-linked glycosylation, preventing the correct folding of N-linked glycosylated proteins, leading to ER stress. Unexpectedly, we found that the *sse1*Δ strain shows significant fitness during ER stress induced by optimal concentrations of Tm, indicating an important regulatory role of Sse1 during ER stress. We further found that the tunicamycin resistance of the *sse1*Δ strain depends on the activation of Ire1-Hac1 mediated ER-UPR signaling. Notably, *SSE1* shows negative genetic interaction with *IRE1* and *HAC1* at physiological conditions in the absence of ER stress, further associating its functions to basal ER-UPR. Moreover, we show that in the absence of *SSE1*, ER-stress-induced changes in ribosome organization are different. The extent of polysome to monosome transition following Tm-induced ER stress is far less prominent in *sse1*Δ cells compared to WT cells. These inefficient alterations in ribosome organization lead to altered kinetics of synthesis of UPR-induced proteins in *sse1*Δ strain where we show that the response to ER stress is quicker and short-lasting compared to WT cells. Importantly, ER-stress-induced cell division arrest as observed in WT cells is evaded in *sse1*Δ strain during Tm-induced ER stress indicating an important role of Sse1 in suspending the cell division following ER stress. Thus, we show that Sse1 plays a key role in maintaining ER protein homeostasis and stress-induced cell division arrest after Tm-induced ER stress. In conclusion, Sse1 despite being a cytosolic chaperone plays a crucial role in deciding cellular fate during global ER stress induced by tunicamycin.

## Materials and methods

### Yeast strains and associated mutants

We used the yeast (*Saccharomyces cerevisiae*) strain BY4741 (*MATa his3Δ1 leu2Δ0 met15Δ0 ura3Δ0*), as our WT and background strain for specific deletion strain. All the *sse1*-double mutants were generated on the commercially available yeast knockout strains from the YKO library employing homologous recombination by using *sse1*-locus-specific *His3MX6* cassette, which was PCR amplified using pFA6a-His3MX6 plasmid as template and primers containing *sse1*-flanking and *His3MX6* sequence. The other yeast strain which we used is *yMJ003* containing the genotype *MATα his3Δ1 leu2Δ0 met15Δ0 ura3Δ0 LYS+ can1Δ::STE2pr-spHIS5 lyp1Δ::STE3pr-LEU2 cyh2 ura3Δ::UPRE-GFP-TEF2pr-RFP-MET15-URA3*. Various plasmid purification and yeast transformation were carried out using standard protocols.

### Yeast culture and growth assay

WT yeast and different deletion strains from the yeast knockout library of the BY4741 background were grown in the YEPD (1% Yeast extract, 2% Peptone, and 2% Dextrose) for overnight to get a saturated culture and the next day a secondary culture was inoculated at 0.1 OD_600_. To perform the drop-dilution assay, secondary cultures of specific yeast strains were grown till the mid-log phase (0.4–0.6 of OD_600_), and serially diluted and spotted on different media plates as mentioned in each experiment. For keeping plasmids, synthetic media with particular auxotrophic selection were used. Liquid growth assay was done using the Bioscreen instrument (Clover Biotech).

### RNA extraction, cDNA synthesis, and *HAC1* mRNA spliced variant determination

Respective yeast strains were grown in suitable media with or without tunicamycin and then the cells were flash-frozen in liquid nitrogen. One milliliter of TRIzol was subsequently added to the flash-frozen cells and was allowed to defrost on ice. After resuspending the cells, 300 mg of acid-washed glass beads (from Sigma-Aldrich, Cat No. G8772) were added. Next, the cells were lysed using Bead-Ruptor (Omni-International) with the speed settings set at 5 units and the total duration of bead-beating was 5 min with intermittent 1 min of incubation on ice after every 1 min of a bead-beating cycle. Then, 200 µl of chloroform (Sigma Aldrich, Cat. No 288306) was added to the cell lysate followed by 15 s of vortexing and then incubated at room temperature for 5 min. Next, the samples were centrifuged at 14,000 RPM for 5 min at 4°C. The supernatant was transferred to a fresh Eppendorf tube and re-extracted with 400 µl of chloroform. Following this step, the supernatant was recovered to a fresh tube, and 500 µl of isopropanol (MP Biochemicals, Cat No 194007) was added and incubated on ice for 15 min to precipitate the RNA. The mixture was pelleted by centrifugation at 14,000 RPM for 5 min at 4°C, and the pellet was washed twice with 1 ml of 70% molecular grade ethanol. The RNA pellet was then air-dried for 15–20 min and resuspended in RNase-free water. For cDNA synthesis, 1 µg of total RNA was mixed with 2 µl of d(T)_23_VN (50 µM) and 1 µl of dNTP min (10 mM) and the reaction volume was made up to 10 µl with sterile water. The mixture was denatured at 65°C for 5 min followed by snap freezing on ice and then the following components were added to the mixture: 4 µl of 5× ProtoScript II Buffer (NEB Cat No. B0368S), 2 µl of 0.1 M DTT (NEB Cat No. B1034A), 1 µl of ProtoScipt II RT (NEB Cat No. M0368S), 0.2 µl of RNase inhibitor (Roche Diagnostics Gmbh Cat No. 03335399001), and 2.8 µl of nuclease-free water. Then, the total cDNA synthesis reaction was incubated at 42°C for 1 hr and then incubated at 65°C for 20 min to deactivate the enzyme. Following this, the newly synthesized cDNA was used as a template in PCR amplification reaction to identify the *HAC1* mRNA splice variants using the following primers: forward primer—5′ CACTCGTCGTCTGATACGTTCACACC 3′, and reverse primer—5′-CATTCAATTCAAATGAATTCAAACCTG-3′. Densitometry was performed using the freely available software from NIH called FIJI, an updated version of the software, ImageJ.

### Western blot

Primary cultures of respective yeast strains were grown overnight. The next day, from the primary culture, the secondary culture is inoculated at 0.1 OD_600_, and the culture was allowed to grow till the mid-log phase (0.4–0.6 OD_600_). Then according to the experimental setup, specific treatments were given and the cells were allowed to grow till the time point set up by the experimental plan. Following treatment, cells were harvested at 8,000 RPM for 5 min. Next, the cell pellet was resuspended in 0.6 ml of 0.3 M NaOH and was incubated for 10 min. Following this, cells were harvested at 4,000 RPM for 1 min. Next, cells were resuspended in 0.3 ml of yeast protein storage buffer (10 mM Tris-HCl, 1 mM EDTA, 150 mM KCl, 1 mM PMSF), and to that 300 mg of acid-washed glass beads (from Sigma-Aldrich, Cat No. G8772) were added. After that, the cells were ruptured with Bead-Ruptor (from Omni-International) with the speed settings set at 3 units and the total time duration was 12 min with intermittent 3 min of incubation on ice after every 3 min of the bead-beating cycle. Next, the cell lysate was spun at 14,000 RPM at 4°C for 30 min. Then, the supernatant was transferred to a separate microfuge tube. Following this, after normalizing the protein concentrations with the BCA protein estimation method, samples were loaded in a 10% SDS-PAGE gel. The western transfer was done using BioRad TransBlot Turbo with Voltage set at 20 V, current set at 1.5 A and time set at 30 min. To visualize equal protein loading, stains like Ponceau S or Amido Black were used along with stain-free UV inducible visible protein adducts were used which do not affect any of the downstream processes like western blotting. Then, the blot was transferred to a blocking solution consisting of 5% BSA in TBST and kept at a slow rocker for 2 hrs at room temperature. After 3 washing steps of the highest rocker speed for 8 min each, the blot was transferred to an anti-Kar2 or anti-Sse1 primary antibody solution with 1:5000 dilutions in 5% BSA in TBST and incubated overnight at 4°C. After that, following three washing steps at the highest rocker speed for 8 min each, the blot was transferred to an HRP-conjugated secondary antibody solution (1:5000 dilution) in TBST and incubated for 2 hrs at 4°C. Following this, 3 washing steps of the highest rocker speed for 8 min were done, and the blot was taken for electro-chemiluminescence and appropriate exposure with X-ray films or in the gel documentation system. The densitometry was performed using the freely available software from NIH called FIJI, an updated version of the software, ImageJ.

### Extraction of total ribosomal fraction

Yeast strains were grown and treated according to the experimental protocol, then the cells were centrifuged at 8,000 RPM for 5 min. Next, cells were washed twice with 0.9% ice-cold potassium chloride, and the cells were collected by centrifugation at 8,000 RPM for 5 min. Cells were then resuspended in buffer A (20 mM Tris-HCl pH 7.5 at 4°C, 5 mM magnesium acetate, 50 mM potassium chloride, 10% glycerol, 1 mM phenyl methyl sulfonyl fluoride, 1 mM 1,4-dithioerythritol) following the 1 g/ml concentration rate, following which 300 mg of acid-washed glass beads were added to the solution. After these cells were ruptured with Bead-Ruptor (from Omni-International) with the speed settings set at 3 units and the total duration of bead-beating was 12 min with intermittent 3 min of ice incubation after every 3 min of rupture cycle. Next, the cell lysates were centrifuged at 16,900 RCF for 30 min. The resulting supernatant was mixed with buffer B (20 mM Tris-HCl pH 7.5 at 4°C, 5 mM magnesium acetate, 0.5 M potassium chloride, 25% glycerol, 1 mM phenyl methyl sulfonyl fluoride, 1 mM 1,4-dithioerythritol) in the ratio of 3:1, respectively, and then the solutions were centrifuged at 1,000,000 RCF at 4°C overnight (approximately 18 hrs) in the fixed angle rotor of Beckman Coulter Ultima Max XP ultracentrifuge. After that, the supernatants were aspirated, and the pellets were washed twice with buffer C (50 mM Tris-HCl pH 7.5 at 4°C, 5 mM magnesium acetate, 50 mM ammonium chloride, 0.1 mM phenyl methyl sulfonyl fluoride, 0.1 mM 1,4-dithioerythritol, 25% Glycerol), and the ribosomes were resuspended in 200 μl of buffer C using a glass rod to gently disrupt the pellet. The solutions were mixed gently for 30 min at 4°C, following which the solutions were transferred to microfuge tubes and centrifuged at 16,900 RCF for 5 min. Next, supernatants were diluted to 3 ml using 0.5 M potassium chloride and then layered over 1 ml of buffer C along with 25% glycerol and centrifuged at 3,00,000 RCF for 4 hrs. After that, the supernatants were aspirated, and the pellets were washed twice with buffer C and again resuspended in 200 µl of buffer C. The solutions were allowed to get mixed again for 30 min at 4°C followed by centrifugation at 16,900 RCF for 5 min. The resulting supernatants containing the ribosomes were transferred to a fresh tube, quantified and stored at −80°C for future use. For visualizing the proteins associated with the ribosomal fractions, RNA normalized samples were mixed with SDS loading dye and loaded onto SDS PAGE gel for Coomassie Blue staining along with western blots that were performed as described earlier.

### UPR induction measurement by flow cytometry

The *yMJ003* strain that we used as the WT and the query strains for *sse1*Δ and *sse2*Δ made in the same background, all of them carry the GFP-tagged UPRE reporter to signify the ER-UPR activation. Here, we cultured the wild-type (WT) cells along with *sse1*Δ and *sse2*Δ in YPD media overnight at 30°C which served as the primary cultures. From those the secondary cultures were inoculated at 0.1 OD_600_ and allowed to grow till 0.4–0.6 OD_600_, then 2.5 µg/ml tunicamycin was added to the cultures to mount ER-UPR and allowed to grow till the time point according to the experimental setup. For flow cytometric measurements, we used the CytoFlex S flow cytometer (Beckman Coulter); the UPRE-GFP fluorescence was detected using the FITC channel. In the case of each strain and each data point, 50,000 individual cells were recorded. The kinetic measurements with 2 hrs interval were captured according to experimental protocol, and the acquired data were analyzed as represented in the Results section.

### Evaluating the real-time protein translation status of UPR target genes by flow cytometry

Here, we took the UPR target genes with a GFP tag from the GFP-tagged library of yeast, and using individual GFP-tagged strains as a background, we deleted *SSE1* using the *URA3* cassette. Thus, the GFP-tagged strains and the *sse1*Δ would serve as the WT and mutant (absence of *SSE1*) strain, respectively. The above strains were used in culturing the primary cells that grew overnight. From that, the secondary cultures were inoculated at 0.1 OD_600_ and were grown till 0.4–0.6 OD_600_, following which 2.5 µg/ml tunicamycin was added along with keeping an untreated control for each WT and mutant strain. GFP fluorescence of the strains was measured at 2 h interval till the time of the experimental setup which signifies the real-time expression levels of their respective GFP-tagged proteins. We used the CytoFlex S flow cytometer (Beckman Coulter) and the GFP fluorescence was detected using the FITC channel. In the case of each strain and each data point, 50,000 individual cells were recorded. After the acquisition, the data were processed and represented as shown in the Results section.

### Polysome profiling

Specific yeast strains were reinoculated in the secondary cultures at 0.1 OD_600_ from the primary cultures and allowed to grow till 0.4–0.6 OD_600_. After that, the desired treatments were given according to the experimental setup. After the treatment was done, cycloheximide (CHX) (50 µg/ml) was added to the media and incubated on ice for 5 min. Then, the cells were harvested at 2,500 RCF for 10 min at 4°C. Then, the cells were resuspended in lysis buffer (50 mM Tris pH—7.5, 150 mM NaCl, 30 mM MgCl_2_, 50 μg/ml CHX) and lysed with the bead beater with the settings 15 s On and 30 s Off for 10 cycles. In between the cycles, the samples were incubated on ice. After this, the cells were centrifuged at 4,000 RPM for 5 min at 4°C and then the supernatants were collected in a fresh tube and spun at 9,200 RPM for 10 min at 4°C. After this, the total RNA content of each strain was normalized by taking absorbance at 260 nm in Nanodrop to proceed with ultracentrifugation. From each, the strain equal amount of RNA (Here 10 absorbance units at 260 nm) should be loaded to the 7–47% continuous sucrose gradient (50 mM tris acetate pH—7.5, 250 mM sodium acetate, 5 mM MgCl_2_, 1 mM DTT and 7, 17, 27, 37, and 47% sucrose) and then centrifuged in Beckman Coulter SW32Ti rotor at 1,65,000 RCF for 4 hrs at 4°C. After the ultracentrifugation, all the samples were fractionated using the ISCO gradient fractionator with the UV detector sensitivity set to 0.5 or 1.0 (the sensitivity should be optimized according to the initial load and the peaks in the profile).

### CLICK chemistry

We used CLICK chemistry to assess the new protein synthesis rate under normal and stressed conditions in various yeast strains. The specified strains required by the experimental setup were reinoculated in secondary cultures at 0.1 OD_600_ from the primary cultures and allowed to grow till 0.4–0.6 OD_600_ in synthetic complete media. After that cells were collected at 5,000 RPM for 5 min, washed with sterile water and then centrifuged at 5,000 RPM for 5 min. Then resuspend the cells in SD-AHA (Standard media where Methionine is replaced with L -azidohomoalanine) and treated with tunicamycin as per the experimental setup. Then the cells were harvested at 8,000 RPM for 5 min, washed with sterile water and then centrifuged at 8,000 RPM for 5 min following which the supernatant was discarded. Then to permeabilize the cells they were resuspended in 53% (v/v) molecular grade absolute ethanol in 1× PBS and incubated in an incubator shaker set at 14.8°C with 200 RPM shaking for 40 min. After that the cells were collected by centrifugation at 8,000 RPM and the supernatant was discarded. The cells were then incubated with 1 ml of CLICK reaction cocktail (2 M Tris-pH8.5, 50 mM Copper Sulphate, 1 µg/ml Alexa-Fluor 488 alkyne, and 0.5 M Ascorbic acid) for 30 min at room temperature. Following that the cells were collected at 8,000 RPM for 5 min washed with 1 ml of 1× PBS and then resuspended in 300 µl of 1× PBS and proceeded for flow cytometry as well as confocal mnicroscopy analysis.

### Statistical analysis

Descriptive statistics for all the measurements that are used for plotting the graphs are expressed as mean ± standard error of the mean (SEM). For calculating the statistical significance, we performed an unpaired *t*-test and one-way ANOVA. If one-way ANOVA yielded a significant difference, then we performed Tukey's test as a post hoc analysis for pairwise comparisons. We set the significance threshold α at 0.05 for all statistical testing that was performed assuming equal variance and the significances were calculated at the two-tailed level. In cases where multiple tests were performed Bonferroni Correction was used to account for the family-wise error rates. The calculated *P*-values were signified as stars in the plots according to the following manner: “ns” meaning *P* > 0.05, “*” meaning *P* ≤ 0.05, “**” meaning *P* ≤ 0.01, “***” meaning *P* ≤ 0.001, and “****” meaning *P* ≤ 0.0001, respectively. For mass spectrometry data analysis, the fold change analysis was done on the raw expression values; following which the fold change values were transformed to a logarithmic scale having base 2 and termed as “Log_2_-Fold Change”. The statistical significance of the expression values was calculated using unpaired *t*-tests assuming equal variance on the Log_2_ transformed expression values. The resulting *P*-values were transformed to a negative logarithmic scale having base 10 and termed as “−Log_10_(*P*)”. Finally, the volcano plots were created using the “Log_2_-Fold Change” values on the X-axis and the “−Log_10_(*P*)” values on the Y-axis.

Other methodologies have been described in the Supplementary Methods.

## Results

### The absence of Sse1 confers tunicamycin resistance to yeast

**Fig. 1.**
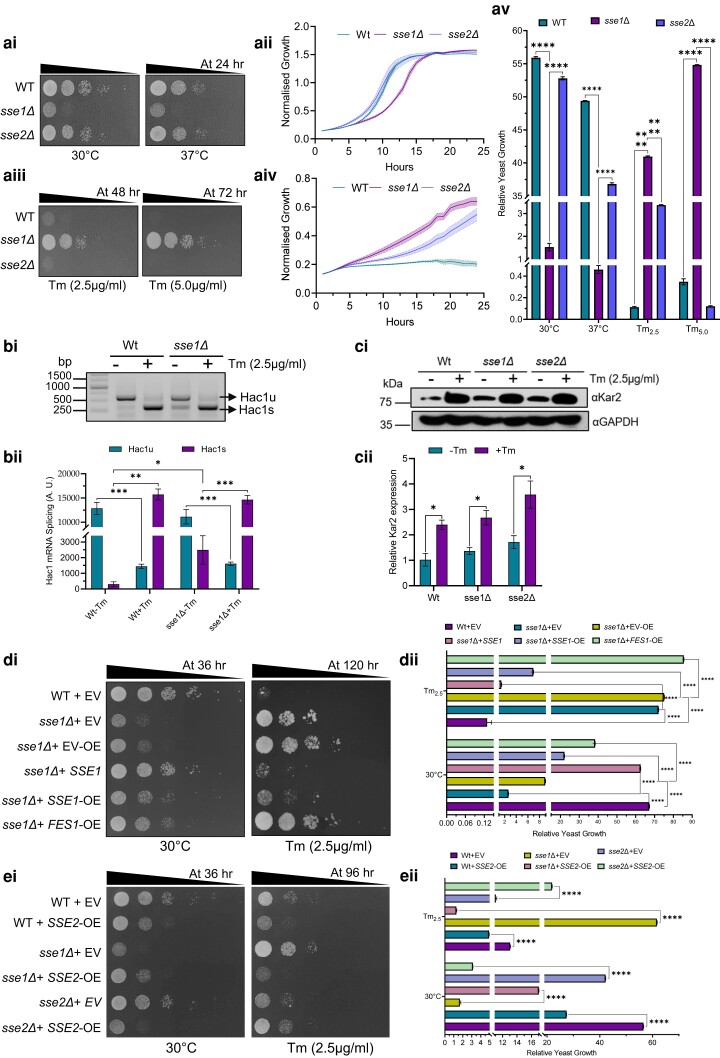
*SSE1* deletion imparts resistance to tunicamycin-induced ER stress in yeast. a) (ai) Yeast growth assay by serial drop dilutions using the strains WT (BY4741), *sse1Δ*, and *sse2Δ* in YPAD plates at permissive temperature (30°C) (left panel) and under heat stress (37°C) (right panel). The triangles above each panel indicate the increasing dilutions. The time mentioned in hours represents the time of incubation before taking the image of the plates. aii) Growth curves in liquid YPD media at permissive temperature (30°C) of the yeast strains used for drop-dilution assay in panel (ai). The normalized growth is plotted as line plot for each strain with shaded area representing the error range for the measurements. (aiii) Similar to panel (ai), a drop-dilution assay was done in presence of ER stressor Tunicamycin (Tm); in two concentrations 2.5 µg/ml and 5.0 µg/ml sufficient to elicit ER-UPR. Longer time of incubation than panel Ai is used here before capturing the pictures of plates as spots appear at later time points due to extremely slow growth of yeast in presence of Tm. (aiv) Growth curves of three strains used in (aiii) in liquid media in presence of tunicamycin (2.5 μg/ml) in YPD are shown as stated earlier in (aii). (av) The spots of the strains from the (ai) and (aiii) panel drop-dilution assays were quantified using densitometry and were plotted as a bar plot with whiskers representing SEM as shown in the right panel (*n* = 3). Statistical significance was calculated using unpaired *t*-tests and the significant pairs were plotted in the graph (all comparisons had *P* < 0.0001, and they were significant even after Bonferroni Correction). b) (bi) The presence of *HAC1* mRNA splice variants was checked from untreated yeast strains (WT, *sse1*Δ) and the same strains after treatment with tunicamycin (2.5 µg/ml) by synthesizing the cDNAs followed by PCR amplifications with the help of specific primers. (bii) The band intensities were quantified using densitometry and were plotted as a bar plot with whiskers representing SEM as shown in the bottom panel (*n* = 3). Statistical significance was calculated using unpaired *t*-tests and the significant pairs were plotted in the graph (Hac1u-WT-Tm/Hac1u-WT + Tm, *P* = 0.0008, ***; Hac1s-WT-Tm/Hac1s-WT + Tm, *P*-value = 0.0002, ***; Hac1u-sse1Δ-Tm/Hac1u-sse1Δ+Tm, *P* = 0.0033, **; and Hac1s-sse1Δ-Tm/Hac1s-sse1Δ+Tm, *P* = 0.0006, ***). All the above pairwise comparisons are significant even after Bonferroni correction with the only exception being Hac1s-WT-Tm/Hac1s-sse1Δ-Tm (one-tailed *P* = 0.0383, *) showed marginal significance and plotted in the graph. c) (ci) Western blot showing the distinct increase in Kar2 (ER-resident Hsp70 and ER-UPR marker) levels in response to ER stress by optimum (2.5 µg/ml) concentration of Tm signifying proper mounting of ER-UPR. GAPDH was used as the loading control. cii) The bands were quantified by densitometry and were plotted as a bar plot with whiskers representing SEM in the bottom panel (*n* = 3). Statistical significance was calculated using unpairedt-tests and the significant pairs were plotted in the graph (WT-Tm/WT + Tm, *P* = 0.0112, *; *sse1*Δ-Tm/*sse1*Δ+Tm, *P* = 0.0142, *; and *sse2*Δ-Tm/*sse2*Δ+Tm, *P* = 0.0347, *). The above pairwise comparisons, except the last pair (*sse2*Δ-Tm/*sse2*Δ+Tm), are significant even after Bonferroni correction. di) Yeast growth assay by serial drop dilutions using the strains wild-type (BY4741), *sse1Δ* transformed with either the empty plasmid vectors (EV or vector control) or the plasmids expressing the Sse1 protein either at endogenous level (from pRS315 plasmid) or overexpressed (from pJV340 plasmid) for complementation assay. Along with Sse1, Fes1, a second cytosolic nucleotide exchange factor (NEF) is also overexpressed from the pJV340 plasmid. The drop-dilution assay was performed using the strains wild-type (BY4741) + pRS315 (empty vector, EV), *sse1Δ* + pRS315 (empty vector, EV), *sse1Δ* + pJV340 (empty vector over expression, EV-OE), *sse1Δ* + pRS315—Sse1, *sse1Δ* + pJV340—Sse1, and *sse1Δ* + pJV340—Fes1 in SD-Leu (synthetic media with Dextrose without leucine) agar plates at permissive temperature (30°C), and in presence of the optimal tunicamycin (2.5 µg/ml) concentrations. The triangles above each panel indicate the increasing dilutions. The time mentioned in hours represents the time of incubation before taking the image of the plates. dii) The spots of the strains from the Di panel drop-dilution assays were quantified using densitometry and were plotted as a bar plot with whiskers representing SEM as shown in the right panel (*n* = 3). Statistical significance was calculated using unpaired *t*-tests and the significant pairs were plotted in the graph (all comparisons had *P* < 0.0001, and they were significant even after Bonferroni Correction). ei) Similar complementation assay using the *SSE2* overexpressing plasmid taken from yeast overexpression library. The strains were grown in SR-Ura + 1% galactose plates at the indicated temperatures and with tunicamycin (right panel). eii) represents the quantification of the spot densities from (ei). Bars were plotted as described in the (dii) panel.

To understand the possible roles of Sse1 and overall Hsp110 molecular chaperones during ER stress, we utilized the well described ER stressor tunicamycin (Tm). We checked the growth phenotype of deletion strain of *SSE1* (*sse1*Δ) during Tm-induced ER stress and other common proteotoxic stresses of varied origin. We kept the wildtype (WT) yeast strain (BY4741) and the deletion strain of *SSE1* paralog, *SSE2*, (*sse2*Δ strain) (Mukai *et al*. 1993) as controls. The *sse1*Δ strain exhibits a prominent growth phenotype at a permissive temperature (30°C) in the absence of any additional stress in comparison to WT and *sse2*Δ strains (Fig. 1ai, left panel; Fig. 1aii and v). A similar phenotype of the *sse1*Δ strain is also observed during heat shock at 37°C (Fig. 1ai, right panel; Fig. 1av). In stark contrast, in the presence of Tm stress, *sse1*Δ strain exhibits a significant fitness in comparison to the WT or *sse2*Δ strains (Fig. 1aiii and iv). In presence of other proteotoxic stress conditions like oxidative stress (induced by H_2_O_2_), protein translation block (induced by CHX or by a limited supply of carbon source), general protein misfolding stress induced by L -azetidine-2-carboxylic acid (AZC), Hsp90 inhibition (by geldanamycin), DNA damage, and mitochondrial stress by ethidium bromide and by CCCP (mitochondrial oxidative phosphorylation uncoupler), or to reducing stress by DTT, *sse1*Δ strain does not exhibit any such fitness (Supplementary Fig. 1a). The deletion strain seems more sensitive to stresses like CHX or H_2_O_2_ treatment compared to untreated cells (Supplementary Fig. 1a and b). Although DTT is quite regularly used as an ER stressor, we did not find any fitness of *sse1*Δ to DTT treatment (Supplementary Fig. 1a, lower left most panel). We reasoned that as DTT apart from imparting a generalized reductive stress, also produces reactive oxygen species (ROS) and in turn imparts oxidative stress during chronic treatment as shown previously (Maity *et al*. 2016), the use of DTT exclusively as an ER stressor is difficult to justify. We also show that *sse1*Δ is more sensitive to treatments with common ROS-producing agents like H_2_O_2_ which may explain its sensitivity towards DTT stress.

Next, the efficient induction of ER-UPR by Tm in the same concentration as used for checking the growth phenotype, was confirmed by significant splicing of *HAC1* mRNA (Fig. 1bi and ii) and overexpression of ER-resident Hsp70 chaperone, Kar2 (yeast ortholog of human BiP protein) (Fig. 1ci and ii). The specificity of the Tm-resistance phenotype of the *sse1*Δ strain is further confirmed by a complementation assay by expressing *SSE1* under its native promoter by a centromeric plasmid in the *sse1*Δ strain (Fig. 1di). Expression of *SSE1* in *sse1*Δ strain reverted the sensitivity to Tm-induced ER stress like WT yeast cells (Fig. 1di, right panel and Fig. 1dii). Importantly, overexpression of *SSE1* successfully restored the Tm-sensitivity similar to the endogenous level of expression although overexpression of another cytosolic NEF, *FES1* could not complement the phenotype (Fig. 1di, right panel). These data indicate that although *FES1* overexpression can rescue the synthetic lethal phenotype *sse1*Δ-*sse2*Δ double deletion strain (lacking NEF activity of Hsp110s) as shown previously (Kumar *et al*. 2020), the role of Sse1 during ER stress cannot be accomplished by Fes1 indicating a possible non-NEF additional function of Sse1 during Tm-induced ER stress. In contrast, overexpression of *SSE2* in *sse1*Δ strain leads to reversal of Tm-sensitivity indicating a Hsp110-specific role during ER stress (Fig. 1ei, right panel, Fig. 1eii). We reiterate that the physiological level of Sse2 is not sufficient to accomplish Sse1's function during ER stress, and the Tm-sensitivity of *sse1*Δ strain, like WT strain, is regained only after *SSE2* overexpression. Moreover, expression of the ATP hydrolysis-deficient mutant of Sse1 (K69Q) in *sse1*Δ cells restored the Tm-sensitivity like WT-Sse1 while the ATP-binding-deficient mutant (Sse1G205D) could not complement the phenotype like WT-Sse1 (Supplementary Fig. 1c, right panel). Another mutant (Sse1G233D) which is reported to be deficient in interaction with Ssa1 and ATP-binding (Shaner *et al*. 2004, 2005), also could not complement the phenotype like WT-Sse1. These data indicate the importance of ATP-binding and not ATP-hydrolysis by Sse1 for its function, during ER stress. Importantly, the *sse1*Δ strain does not exhibit any fitness when treated with subcritical concentrations of Tm (Supplementary Fig. 1d) which is not adequate to mount ER-UPR (Supplementary Fig. 1ei, ii and fi, ii). This finding indicates that tunicamycin resistance of the *sse1*Δ strain is dependent on the efficient mounting of ER-UPR. As we found Tm-resistance of *sse1*Δ exclusively at higher concentrations of the stressor that is sufficient to mount ER-UPR but not at the lower concentration, we checked a panel of Tm concentrations to understand the concentration range where the fitness is observed. We show that above 1 µg/ml of concentrations Tm (usually 2 µg/ml or higher concentrations of Tm are used to elicit ER-UPR in yeast), *sse1*Δ strain shows strong fitness against this ER stressor (Supplementary Fig. 2a). Furthermore, to check any adaptive changes in the glycosylation status of proteins in *the sse1*Δ strain, we specifically captured the glycosylated proteins using concanavalin A (ConA) from both *sse1*Δ and WT cells. There was no visible change in the glycosylated proteins in the *sse1*Δ strain (Supplementary Fig. 1g) ruling out the possibility of adaptive enhanced glycosylation or proteins in the *sse1*Δ strain which may confer growth fitness to this strain during Tm-induced ER stress.

### *SSE1* exhibits negative genetic interaction with *IRE1* and *HAC1* and the tunicamycin resistance of *sse1*Δ strain depends on the presence of functional ER-UPR signaling by the Ire1-Hac1 pathway

**Fig. 2.**
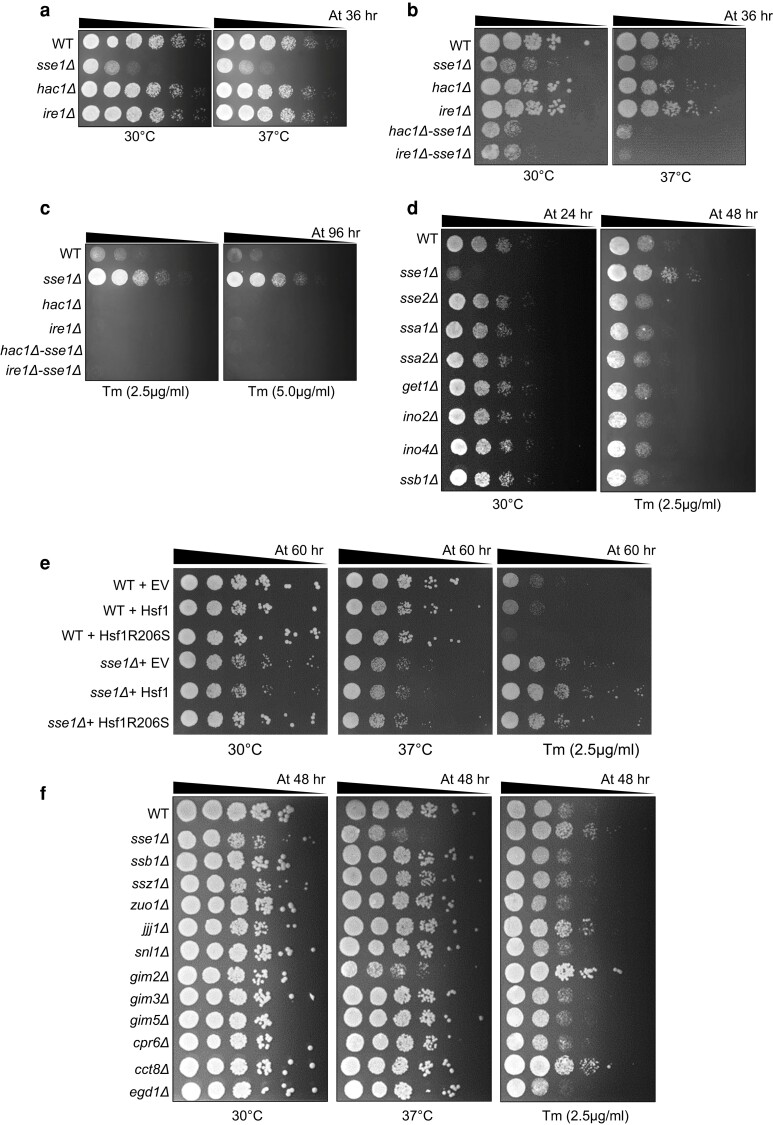
Tunicamycin resistance of *sse1Δ* is dependent on *IRE1-HAC1* mediated ER-UPR signaling and the fitness is attributed to the abrogation of CLIPS function of Sse1 and not to the basal high heat shock response of the strain. a) Yeast growth assay by serial drop dilutions using the strains WT, *sse1Δ*, *hac1Δ*, and *ire1Δ* in YPAD plates at permissive temperature (30°C) and at heat-shock condition (37°C). The time mentioned in hours represents the time of incubation before taking the image of the plates. b) Drop-dilution assay as shown in (a) using the strains WT, and single deletion strains *sse1Δ*, *hac1Δ*, *ire1Δ*, and double deletion strains, *hac1Δ-sse1Δ* and *ire1Δ-sse1Δ* in YPAD plates at permissive temperature (30°C) and at heat-shock condition (37°C). c) The same strains as in (b) were used for drop-dilution assay at optimal concentrations of Tm (2.5 µg/ml and 5.0 µg/ml). (d) Drop-dilution assay of WT, *sse1Δ* and all other single deletion strains of yeast reported to exhibit high basal Heat Shock Response (HSR), (*sse2Δ*, *ssa1Δ*, *ssa2Δ*, *get1Δ*, *ino2Δ*, *ino4Δ*, and *ssb1Δ*) in YPAD plates at permissive temperature (30°C) (left panel), and in presence of ER stressor Tm (2.5 µg/ml) (right panel). e) The effect of constitutive activation of heat shock response (HSR) on Tm-resistance phenotype of WT and *sse1Δ* strains was checked by expressing the Hsf1R206S mutant from plasmid by drop dilution assay. The strains used here are WT (BY4741) + pRS423 (empty vector, EV), WT + pRS423—Hsf1, WT + pRS423—Hsf1-R206S, *sse1Δ* + pRS423 (empty vector, EV), *sse1Δ* + pRS423—Hsf1, and *sse1Δ* + pRS423—Hsf1-R206S in SD-His (synthetic media with dextrose without histidine) agar plates at permissive temperature (30°C), heat stress condition (37°C), and in presence of the optimal tunicamycin (2.5 µg/ml) concentrations. f) Similar to (e), growth assay by serial drop dilutions was performed for the WT, *sse1Δ* and all other single deletion strains for CLIPS proteins apart from Sse1, (*ssb1Δ*, *ssz1Δ*, *zuo1Δ*, *jjj1Δ*, *snl1Δ*, *gim2Δ*, *gim3Δ*, *gim5Δ*, *cpr6Δ*, *cct8Δ*, and *egd1Δ*) in YPAD plates in permissive temperature (30°C) (left panel), heat shock condition (37°C) (middle panel) and in presence of an optimal concentration of tunicamycin (2.5 µg/ml) (right panel). All spots shown in (a–e) were quantified and the quantifications have been shown in Supplementary Fig. 5.

The absence of growth fitness of *sse1*Δ strain at subcritical Tm concentrations hinted towards the necessity of a threshold of ER stress that is sufficient to mount ER-UPR, for gaining fitness against Tm-induced ER stress. As ER-UPR induction is solely dependent on the Ire1-Hac1 pathway in yeast, to understand the Ire1-Hac1 signaling dependence of Tm-resistance of *sse1*Δ strain, we deleted *SSE1* in *ire1*Δ or *hac1*Δ strains to generate the double knockouts of the *ire1*Δ-*sse1*Δ and *hac1*Δ-*sse1*Δ strains. Single deletion strains, *ire1*Δ or *hac1*Δ, grow similarly to WT cells at permissive temperature (30°C) (Fig. 2a, left panel; Supplementary Fig. 5a) as well as during heat stress (37°C) (Fig. 2a, right panel; Supplementary Fig. 5a) indicating no alterations in growth rate of yeast under physiological conditions or even during heat stress in absence of the ER-UPR sensors. Interestingly, the double knockout strains of *ire1*Δ-*sse1*Δ and *hac1*Δ-*sse1*Δ, show synthetic growth defects at a permissive temperature in the absence of any additional stress indicating a negative genetic interaction between *SSE1* and *IRE1* as well as between *SSE1* and *HAC1* (Fig. 2b, left panel and Supplementary Fig. 5b). To the best of our knowledge, any experimental evidence of the genetic interaction between *SSE1* and *IRE1-HAC1* pathway is hitherto unknown in literature and this finding implicates an important role of *SSE1* in ER-UPR. During heat stress at 37°C, the synthetic growth sickness of *ire1*Δ-*sse1*Δ and *hac1*Δ-*sse1*Δ strains is significantly aggravated compared to the *sse1*Δ strain, indicating a stronger genetic interaction with *SSE1* and ER-UPR sensors during heat stress (Fig. 2b, right panel; Supplementary Fig. 5b). Upon treatment with Tm, *ire1*Δ or *hac1*Δ strains could not grow as expected, due to lack of mounting of ER-UPR (Fig. 2c; Supplementary Fig. 5b). Upon deletion of either *IRE1* or *HAC1*, the growth fitness observed for *sse1*Δ during Tm-treatment was completely abolished reiterating the fact that the Tm-resistance of *sse1*Δ strain depends on the presence of functional ER-UPR signaling by canonical Ire1-Hac1 pathway (Fig. 2c, Supplementary Fig. 5b). In sub-optimal concentrations of Tm that are not sufficient to mount ER stress, the phenotypes of *sse1*Δ or *ire1*Δ-*sse1*Δ and *hac1*Δ-*sse1*Δ remained similar to Tm-untreated condition (Supplementary Fig. 2b).

In summary, we show that *SSE1* genetically interacts with the ER-UPR pathway and Tm-resistance of *sse1*Δ strain is dependent on the efficient mounting of Ire1-Hac1 mediated ER-UPR signaling.

### Similar to *SSE1*, the deletion of two other CLIPS members, imparts resistance to tunicamycin-induced ER stress in yeast

It was previously shown that the deletion of many genes including the *sse1*Δ strain, increases the basal heat shock response (HSR) of yeast, *S. cerevisiae* (Brandman *et al*. 2012). Another study also found that HSR alleviates ER stress (Liu and Chang 2008). Thus, to explain the Tm-resistance of the *sse1*Δ strain, we hypothesized that this strain's high basal HSR during ER stress may be responsible for conferring Tm resistance.

To check this, we took the deletion strains of yeast from the Yeast Knockout (YKO) library, which are reported to exhibit high basal HSR, including *sse1*Δ. Among the eight deletion strains (*sse1*Δ, *sse2*Δ, *ssa1*Δ, *ssa2*Δ, *get1*Δ, *ino2*Δ, *ino4*Δ, and *ssb1*Δ) previously reported to show high basal HSR (Brandman *et al*. 2012), none other than *sse1*Δ show any fitness during Tm-induced ER stress (Fig. 2d and Supplementary Fig. 5c). These data indicate that the growth fitness observed for the *sse1*Δ strain is not due to high basal HSR as none of the other deletion strains possessing high HSR exhibit any fitness during Tm-induced ER stress. Furthermore, to check the role of HSR, we expressed the constitutively active mutant of Hsf1 (Hsf1R206S) in WT and *sse1*Δ strains and checked the phenotype (Fig. 2e and Supplementary Fig. 5d). Constitutive activation of HSR by Hsf1R206S in WT did not impart any fitness advantage to Tm-stress at 2.5 µg/ml concentration of the stressor rather it increased the sensitivity to Tm-stress, negating the role of high HSR in Tm-resistance (Fig. 2e, right panel and Supplementary Fig. 5d). In case of *sse1*Δ strain, Hsf1R206S expression leads to mild growth phenotype alleviation at ambient and at heat shock conditions, although there is no additional enhancement of tunicamycin resistance of the *sse1*Δ strain due to constant HSR activation (Fig. 2e, right panel and Supplementary Fig. 5d). This result further confirms Sse1's specific role in cellular response during Tm-induced ER stress which cannot be complemented by activated HSR.

As Sse1 belongs to a class of chaperones termed as CLIPS (Albanese *et al*. 2006) due to its co-regulated expression pattern with cytosolic translation machinery, we hypothesized that deletion of *SSE1* would lead to altered or inefficient protein translation as indicated by enhanced sensitivity of *sse1*Δ strain to translation blocker, CHX (Supplementary Fig. 1bi and ii). Thus, *SSE1* deletion may reduce the incoming protein load to ER which in consequence would help better management of ER stress, leading to the observed fitness. Next, we took the single deletion strains of all the CLIPS (*sse1*Δ, *ssb1*Δ*, ssz1*Δ, *zuo1*Δ, *jjj1*Δ, *snl1*Δ, *gim2*Δ, *gim3*Δ*, gim5*Δ*, cpr6*Δ, *cct8*Δ, and *egd1*Δ) (Albanese *et al*. 2006) and checked the phenotype during Tm-induced ER stress. Interestingly, apart from *sse1*Δ, we observed similar fitness in deletion strains of three other CLIPS, namely *JJJ1*, *GIM2*, and *CCT8* (Fig. 2f and Supplementary Fig. 5e). *CCT8* deletion showed varied phenotypes in the repeat experiments and we did not proceed with it further. Jjj1 is a cytosolic J-domain cochaperone protein of Hsp70 protein Ssa1 and it remains associated with large ribosomal subunits. Jjj1 was shown to be involved in the late stage, cytosolic steps of biogenesis of 60S ribosomal particles (Meyer *et al*. 2007). Gim2 is part of the prefoldin complex which works as cochaperone of the CCT/TRiC chaperonin complex (Geissler *et al*. 1998). Prefoldin plays a crucial role in cytoskeleton assembly by helping the folding of actin and tubulin monomers (Millan-Zambrano and Chavez 2014). So far, there is no evidence of interaction of *SSE1* with two of these CLIPS (Jjj1 and Gim2) in literature, thus to check any genetic interaction between *SSE1* and these three CLIPS, we made double knockouts of *SSE1-JJJ1* and *SSE1-GIM2*. *SSE1* shows strong negative genetic interactions with both *JJJ1* and *GIM2* at permissive temperature (30°C) (Supplementary Fig. 2c, left panel). *SSE1-GIM2* genetic interaction is very strong at permissive temperature and the double deletion shows extreme synthetic sickness with heat stress at 37°C (Supplementary Fig. 2c, middle panel). Interestingly, in the double deletion strains of *SSE1-JJJ1* and *SSE1-GIM2*, Tm-resistance of individual single deletion strains of these CLIPS is abolished. The double knockout strains show similar Tm-sensitivity like wild-type yeast (Supplementary Fig. 2c, right panel). These data indicate that during Tm-induced ER stress, *SSE1* genetically interacts with *JJJ1* and *GIM2* individually and the Tm-resistance of *sse1*Δ depends on intact function of *JJJ1* or *GIM2* and vice versa. To confirm whether these CLIPS work in parallel pathways to Sse1 during Tm-stress, needs further exploration.

In summary, we found that the absence of CLIPS function of Sse1 in *sse1*Δ strain rather than the high basal heat shock response of strain, plausibly imparts resistance to Tm-induced ER stress.

### Sse1 controls the ER-stress-induced changes in cellular protein translation

**Fig. 3.**
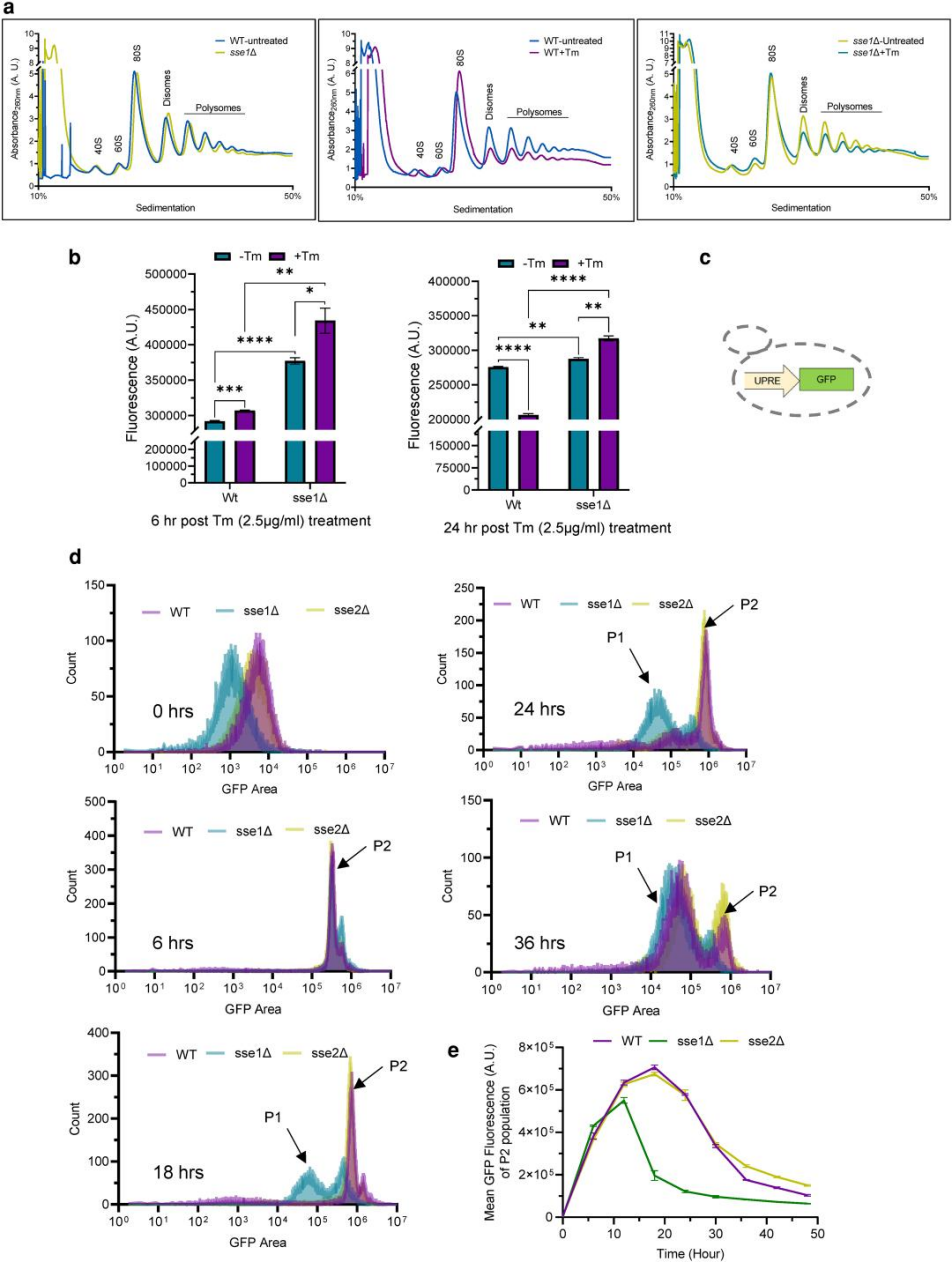
Sse1 plays an important role in modulating tunicamycin-induced ER stress-associated changes in protein translation. a) Polysome profiles are plotted for the WT and *sse1Δ* strains isolated from untreated cells and cells treated with Tm (2.5 µg/ml) and comparison between polysome profiles of (left panel) untreated WT vs. untreated *sse1Δ*, (middle panel) untreated vs. Tm-treated WT cells and (right panel) untreated vs. Tm-treated *sse1Δ* cells are shown. b) The rate of translation of WT, and *sse1Δ* strains in untreated and Tm-treated (2.5 µg/ml) conditions were analyzed using the CLICK-IT chemistry reaction using L -azido-homoalanine (AHA) and Alexa-Fluor 488 alkyne dye. The incorporated fluorescence in newly synthesized proteins was measured in each sample by flow cytometry and was plotted as a bar plot with whiskers representing SEM. The left panel shows the incorporated fluorescence following 6 hrs of Tm-stress in comparison to untreated cells of WT and *sse1Δ* strains. The right panel shows same after 24 hrs of Tm-treatment. Statistical significance was calculated using unpaired T-tests and the pairwise comparison outputs were plotted in the graph (Left panel: WT-Tm/WT + Tm, two-tailed *P* = 0.0003, ***; *sse1*Δ-Tm/*sse1*Δ+Tm, two-tailed *P* = 0.0367, *; WT-Tm/*sse1*Δ-Tm, two-tailed *P* < 0.0001, ****; WT + Tm/*sse1*Δ+Tm, two-tailed *P* = 0.0021, **. Right panel: WT-Tm/WT + Tm, two-tailed *P* < 0.0001, ****; *sse1*Δ-Tm/*sse1*Δ+Tm, two-tailed *P* = 0.0019, **; WT-Tm/*sse1*Δ-Tm, two-tailed *P* = 0.0052, **; WT + Tm/*sse1*Δ+Tm, two-tailed *P* < 0.0001, ****). c) Schematic of the yeast strain YMJ003 (wild type) that serves as a reporter strain for ER-UPR activation. The strain contains the GFP under unfolded protein response element (UPRE) to report for ER-UPR induction. d) Kinetics of ER-UPR activation was measured by measuring UPRE-GFP fluorescence by flow cytometry and shown as overlaid histograms at different timepoints post-Tm-treatment. Each histogram represents the data of the strains WT (YMJ003), *sse1Δ*, and *sse2Δ* (in YMJ003 strain background) at designated time points mentioned in each panel following treatment with Tm (2.5 µg/ml). The shift in the two populations, P2 (UPR-activated population) and P1 (basal state after reversal from UPR-activated state), among the strains are marked accordingly. e) The P2 population's (as shown in d) mean GFP fluorescence intensity over time is plotted in this line plot for the same set of WT, *sse1*Δ and *sse2*Δ cells. The whiskers over each time point value represents SEM.

In the previous section, we have shown that in absence of *SSE1* or individual deletion of the other two CLIPS, *JJJ1* and *GIM2* imparts tunicamycin resistance to yeast cells. Thus, it was interesting to assess any changes in protein translation following Tm-induced ER stress, especially in the absence of Sse1. To capture the status of cellular translating ribosomes, we performed the polysome profiling of WT and *sse1*Δ strain in the absence of any external stress and following imparting Tm-induced ER stress. In the physiological condition, the polysome profile of WT yeast cells shows a substantial part of total ribosomes in the polysome fraction along with the monosome (80S) fraction (Fig. 3a, left panel). Upon inducing ER-stress by tunicamycin, a significant part of the polysomes is shifted to monosome fraction in WT yeast cells (Fig. 3a, middle panel). In case of *sse1*Δ strain, the polysome profile reveals a similar presence of polysome and monosome fractions (Fig. 3a, left panel). Interestingly, upon Tm-treatment, an equivalent reduction in polysome fraction is not explicitly prominent in the *sse1*Δ strain in contrast to WT cells (Fig. 3a, right panel). These data indicate an important regulatory role of Sse1 during ER-stress-induced reorganization of the cellular translation apparatus. This finding points towards the possibility of unhindered polysome-driven protein translation in *sse1*Δ strain compared to WT cells during ER stress.

To check any difference in protein synthesis status between *sse1*Δ strain and the WT yeast, we measured the synthesis of new proteins by incorporating AHA (L-Azidohomoalanine) for tagging the newly synthesized proteins by Click-IT chemistry (Chang *et al*. 2021). We measured the new protein synthesis status at multiple time points following the induction of ER stress by Tm-treatment using flow cytometry. Interestingly, after 6 hrs of Tm-treatment, the amount of newly translated proteins in *sse1*Δ strain was significantly higher than the WT cells which nicely corroborated with the ribosome profile of these two strains (Fig. 3b, left panel). When we measured the AHA fluorescence post 24 hrs of Tm-treatment, compared to untreated cells, we observed a significant reduction in the incorporated fluorescence in WT cells indicative of a reduction of new protein synthesis (Fig. 3b, right panel). In sharp contrast, Tm-treated *sse1*Δ cells showed significantly higher new protein synthesis compared to untreated cells at the same time points (Fig. 3b, left and right panels). Finally, the comparison of new protein synthesis status between WT and *sse1*Δ strains post 24 hrs of Tm-stress showed a drastic increase in AHA incorporation in *sse1*Δ cells indicative of significantly higher translation of new proteins in *sse1*Δ strain. This trend of new protein synthesis remained similar upon continuing the Tm-stress for 48 hrs (Supplementary Fig. 3a).

As AHA incorporation indicates all new protein translation, to exclusively measure the formation of UPR-induced proteins following ER stress, we employed the previously described UPR-reporter strain, YMJ003 (Jonikas *et al*. 2009; Maity *et al*. 2016). This strain contains genome-integrated enhanced green fluorescence protein (EGFP) which is expressed under the UPR element (UPRE) whenever cells experience ER stress (Fig. 3c) (Jonikas *et al*. 2009; Maity *et al*. 2016). In the background of YMJ003, we deleted *SSE1* and also *SSE2* (as control) which showed similar phenotypes as observed in the case of BY4741 WT background as described in both Figs. 1 and 2 (Supplementary Fig. 2d). When we compared the kinetics of UPR by following the GFP fluorescence by flow cytometry, we observed a very distinctive kinetics of ER-UPR induction in *sse1*Δ strain as compared to WT or *sse2*Δ strains (Fig. 3d and e). In all three strains, we observed the time-dependent appearance of a high-fluorescent population (UPR-activated population denoted as P2 population) after the Tm-stress. After 2 hrs of Tm-treatment, about 20–30% population shifts to the P2 population (Supplementary Fig. 3b) which becomes nearly 100% by 6 hrs in all the strains indicating near complete mounting of ER-UPR (Fig. 3d left middle panel and Supplementary Fig. 3b). Interestingly, for the *sse1*Δ strain, we observed a faster reversal to basal state (P1 population) compared to WT or *sse2*Δ strains (Fig. 3d and e). By 18 hrs post-Tm-treatment, we observed more than 60% of *sse1*Δ cells in the P1 population whereas WT or *sse2*Δ strains showed less than 10% cells in the P1 population (Fig. 3d left lower panel and Supplementary Fig. 3b). By 24 hrs, almost 75% of *sse1*Δ cells shift to the P1 population while only about 18% of the WT or *sse2*Δ cells are present in the P1 population (Fig. 3d right upper panel and Supplementary Fig. 3b). This result nicely indicates a faster reversal from the UPR-activated state for *sse1*Δ strain in comparison to WT strain. The mean GFP intensity plot of the UPR-activated cell population (P2 population) of these 3 strains further indicates a quicker response to ER stress and reversal to the basal state by *sse1*Δ strain as compared to WT or *sse2*Δ strains (Fig. 3e). WT or *sse2*Δ strains show much higher and sustained response to chronic Tm-induced ER stress (Fig. 3e). This result shows the important role of Sse1 in maintaining a prolonged response to ER stress during global ER stress by tunicamycin.

**Fig. 4.**
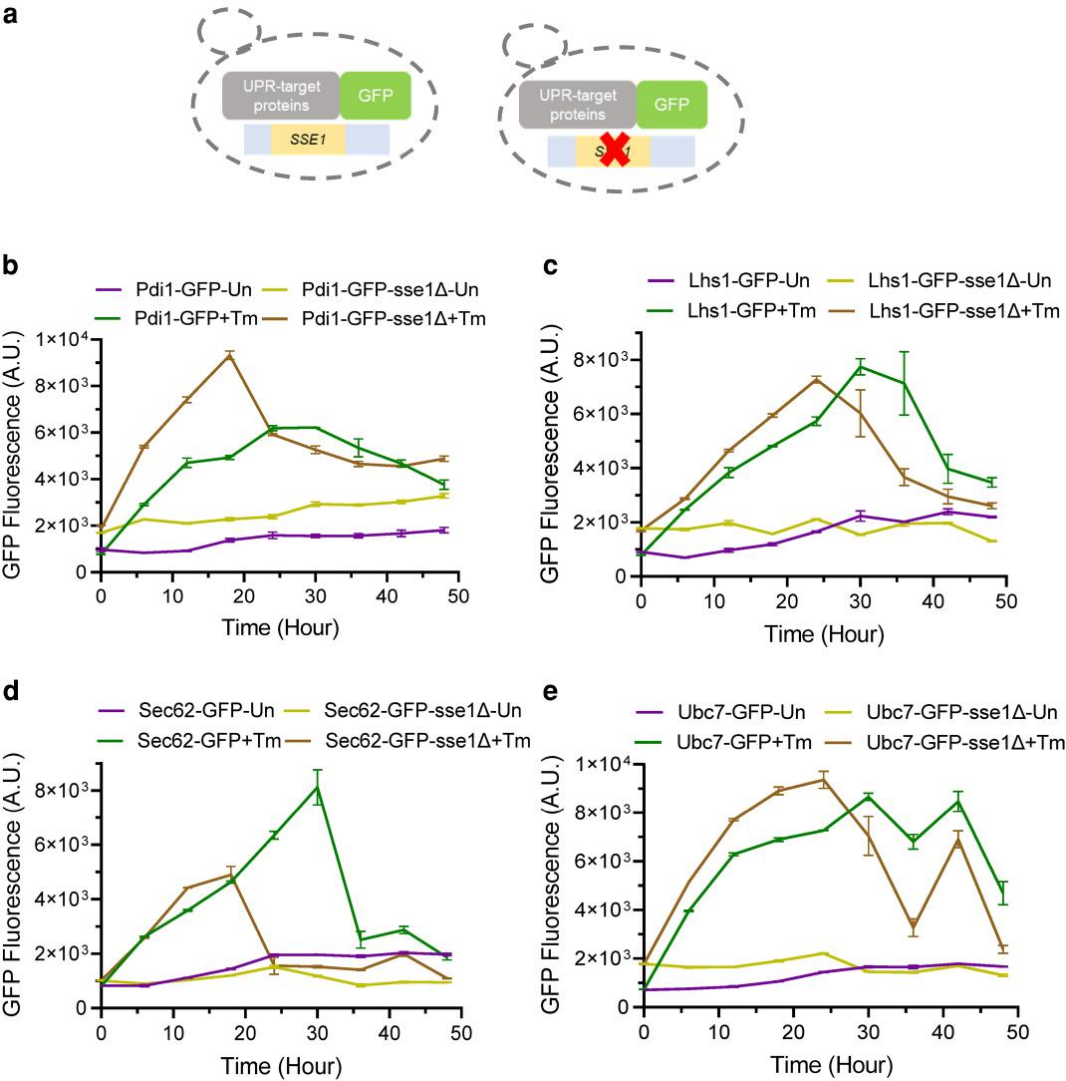
The ER-UPR kinetics is different in absence of Sse1. a) (Left) Schematic of the yeast strains where individual UPR target genes are tagged with GFP, so that their real time translation can be monitored by measuring the GFP fluorescence. (Right) Using these strains as background, *SSE1* was deleted using *URA3* cassette in each of the GFP reporter strain. The left panel strains serve as the WT strain and the right panel strain serve as *sse1*Δ strain. b–e) The GFP-tagged UPR target protein's expression as measured by flow cytometry over time are plotted. The mean GFP fluorescence of the UPR-activated population is plotted as line plots at designated time points following treatment with Tm (2.5 µg/ml). The line plots of the expression of cellular targets of ER-UPR like Pdi1-GFP (b), Lhs1-GFP (c), Sec62-GFP (d), and Ubc7-GFP (e) strains with *sse1*Δ counterparts along with untreated and Tm-treated samples are shown. The whiskers at each time point value represents SEM.

Next, to follow the actual cellular response in terms of synthesis of UPR-responsive proteins following induction of ER stress, we took different GFP-tagged strains of ER-UPR target proteins like Pdi1, Lhs1, Sec62, and Ubc7 to monitor the protein synthesis following induction of ER stress, by flow cytometry (Fig. 4a, left panel). In these GFP-tagged strains, we deleted the genomic copy of *SSE1* (Fig. 4a, right panel). Interestingly, for all ER-UPR targets Pdi1, Lhs1, Sec62, or Ubc7, we observed significant time-dependent increased expression of these proteins following Tm-treatment indicating mounting of ER-UPR followed by a gradual decrease in the protein level indicative of decay of the protein levels and reversal from ER-UPR-activated state to basal condition (Fig. 4b–e). The expression of these proteins remained at a much lower basal level in the untreated cells confirming the suitability of checking the expression levels of these target proteins as reporters of ER-UPR induction by tunicamycin (Fig. 4b–e). Importantly, the corresponding *SSE1*-deleted versions of the GFP-tagged strains showed the highest expression of all these ER-UPR target proteins around 18–22 hrs post-Tm-treatment followed by a decrease in the protein expression to basal level by 30–32 hrs (Fig. 4bi–ei). In contrast, in case of the WT strains, the expression of these proteins peaked at later time points (28–30 hrs post-Tm-treatment) followed by a decay in the protein levels at much later time points (around 40 hrs) (Fig. 4b–e). Similar to the UPRE-GFP reporter, the GFP-tagged ER-UPR target proteins also showed a UPR-activated state (P2 population, data not shown) at higher fluorescence intensity and a lower fluorescent P1 population in the later time points indicative of basal state after reversal from the UPR-activated state (data not shown). Overall, the quick response to ER stress and faster reversal to the basal state from the UPR-activated state by the *sse1*Δ strain in comparison to the WT strain as detected by the UPRE-GFP reporter described in the previous section (Fig. 3d and e) was further confirmed by following the UPR-activated expression of actual cellular UPR-target proteins (Fig. 4b–e).

In summary, we show that in WT cells, in the presence of Sse1, ER-UPR is a sustained process following Tm-induced ER stress. Interestingly, in absence of Sse1, activation of ER-UPR as well as reversal to basal state indicative of restoration of homeostasis following Tm-induced ER stress, is much quicker which can explain the fitness to Tm-stress observed in the *sse1*Δ strain.

### Cellular response to Tm-induced ER stress is distinctly different in the absence of Sse1

**Fig. 5.**
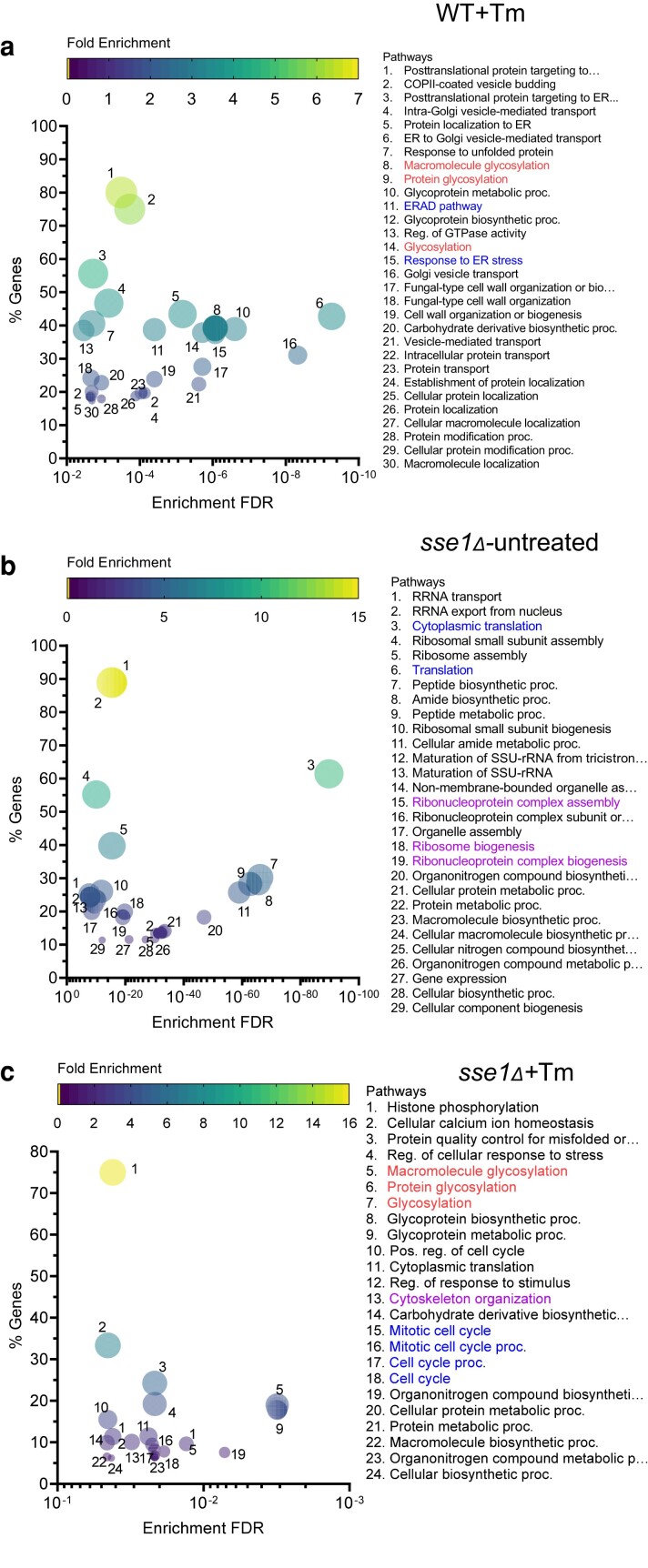
The pathway enrichment analysis of the cellular transcriptome upon tunicamycin stress in WT and *sse1*Δ strains. a–c) Transcriptome analysis of WT and *sse1*Δ strains in untreated condition and after treatment with tunicamycin (2.5 μg/ml) along with other yeast strains of same genetic background as described previously (Narayana Rao *et al*. 2022) was done by RNA sequencing. The outputs were converted to Z scores. Transcripts above and below z-score 2 were considered as differentially upregulated or downregulated, respectively. The multivariate bubble plots showing the enriched upregulated pathways with the attributes—the enrichment false discovery rate (enrichment FDR) on the X-axis, the percentage of genes identified for each of the enriched pathway with respect to the total annotated genes on the Y-axis, the color scale represents the fold enrichment, and the larger bubble size signifies highly enriched and significant pathways. a) Bubble plot representing the pathways enriched in WT cells when treated with tunicamycin (2.5 μg/ml). Bubble plot showing the enriched pathways of the *sse1*Δ strain in untreated condition (b) and tunicamycin (2.5 μg/ml)-treated condition (c). The important enriched pathways in all three cases have been highlighted in different colors.

To understand the cellular response during Tm-stress, we did an RNA sequencing-based transcriptome analysis and a label-free quantitative proteomics analysis of untreated and Tm-treated WT and *sse1*Δ strains. For transcriptome analysis, along with 40 other yeast samples of similar genetic background, WT, and *sse1*Δ strains were subjected to RNA sequencing in untreated and Tm-treated conditions. To identify the genes that are differentially upregulated or downregulated in a particular sample, the Z-score of expression for each gene across all these yeast strains was calculated as described previously (Narayana Rao *et al*. 2022). The genes above or below Z-score 2 at a particular condition were considered as significantly upregulated or downregulated, respectively. Using Z-score analysis of the transcriptomics data, we found 885 genes to be differentially upregulated and 9 genes to be downregulated in the WT strain upon Tm-treatment. In contrast, *sse1*Δ strain showed 415 genes to be upregulated and 115 genes to be downregulated in the untreated condition. Upon Tm-treatment, *sse1*Δ strain showed 370 genes to be upregulated and 14 genes to be downregulated. Next, we did a pathway enrichment analysis of upregulated genes (Fig. 5) found in the WT-treated cells and *sse1*Δ cells in both untreated and Tm-treated conditions. Among the top 30 enriched pathways in Tm-treated WT cells, response to unfolded proteins, macromolecule glycosylation, protein glycosylation, ERAD pathway etc. clearly shows the response to tunicamycin stress and activation of ER-UPR (Fig. 5a and Supplementary Table 2). Many enriched pathways show changes in intracellular protein trafficking and protein localization. In the *sse1*Δ strains, the untreated condition shows a very significant enrichment of ribosome assembly, cytoplasmic protein translation and various biosynthetic pathways (Fig. 5b and Supplementary Table 2). STM1, a protein required for optimal translation during stresses was upregulated in the *sse1*Δ strain in the transcriptomics data (Supplementary Table 4). In the *sse1*Δ Tm-treated transcriptome, we found macromolecule and protein glycosylation, and protein quality control pathways similar to WT-treated cells (Fig. 5c and Supplementary Table 2). Interestingly, we found cytoplasmic translation, cytoskeleton reorganization, and cell cycle pathways to be uniquely enriched in the *sse1*Δ treated cells (Fig. 5c and Supplementary Table 2). Many unique transcription factors related to these upregulated pathways have been shown in the Supplementary Table 4.

**Fig. 6.**
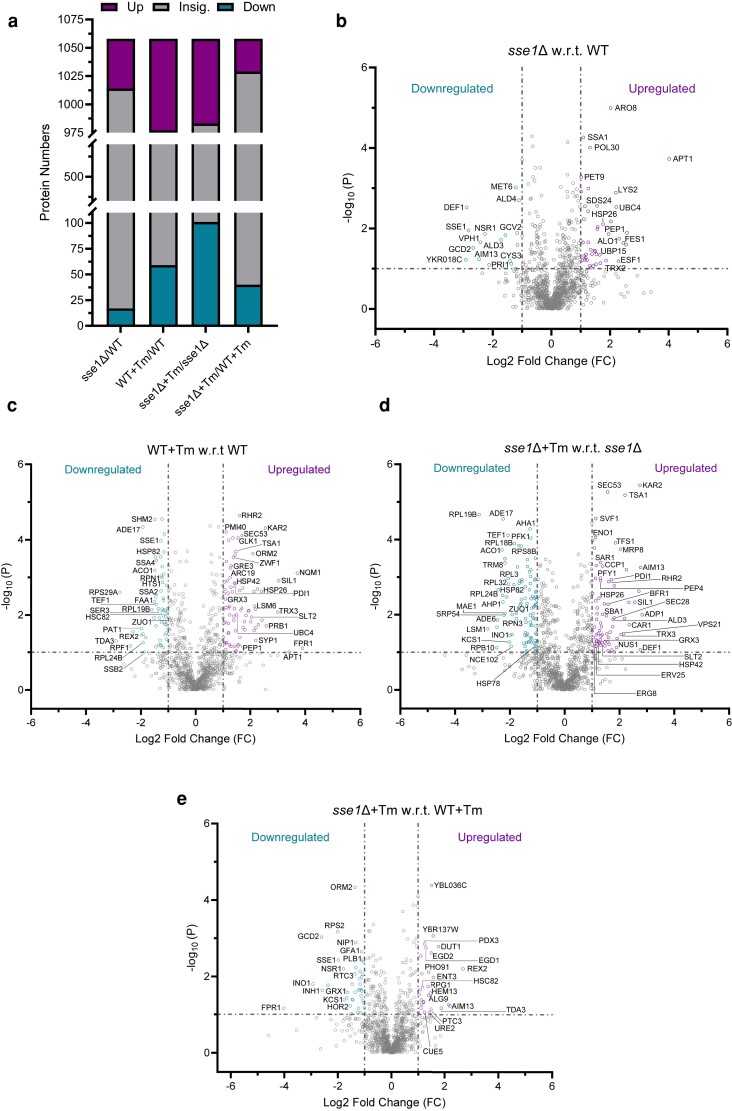
The changes in cellular proteome upon tunicamycin stress in WT and *sse1Δ* strains. a) The total number of proteins identified by quantitative mass spectrometry and the outputs of the statistical analysis of various pairwise comparisons are plotted as composite bar plots showing the total number of proteins that were differentially upregulated, downregulated, and insignificant for each of the comparisons between the *sse1Δ* and WT strain in untreated and after treatment with optimal concentration of tunicamycin (2.5 µg/ml). b–e) Volcano plots showing the differentially expressed (upregulated and downregulated) as well as proteins with insignificant changes in the expression level in the untreated *sse1Δ* strain with respect to the untreated WT strain (B), in the Tm (2.5 µg/ml) treated WT strain with respect to the untreated WT strain (C), in the Tm (2.5 µg/ml) treated *sse1Δ* strain with respect to the untreated *sse1Δ* strain (D) and in Tm (2.5 µg/ml) treated *sse1Δ* strain with respect to Tm (2.5 µg/ml) treated WT strain (E).

Next, by quantitative proteomics analysis, 1,058 proteins were detected in all three replicates of WT and *sse1*Δ Tm-treated and untreated samples which were analyzed further (Supplementary Table 3). Comparison of protein levels of untreated *sse1*Δ cells with respect to WT cells at basal condition (no stress) revealed 44 and 17 proteins to be differentially upregulated and downregulated, respectively (Fig. 6a and Supplementary Table 3). Rest 997 proteins did not show any significant changes in the expression level. Among the differentially upregulated proteins in untreated *sse1*Δ cells with respect to WT cells, cytosolic Hsp70, Ssa1, small heat shock protein Hsp26 and another cytosolic NEF, Fes1, were detected (Fig. 6b). Upregulation of Ssa1, Fes1, and Hsp26 indicates activation of HSR as *sse1*Δ is known to exhibit high basal HSR (Brandman *et al*. 2012). Next, comparison of protein levels of Tm-treated WT cells with respect to untreated WT cells revealed a drastic increase in the number of differentially expressed proteins due to ER stress (Fig. 6c and Supplementary Table 3). Tm-induced stress led to differential upregulation of 81 proteins and downregulation of 59 proteins in the WT strain and changes in the expression of the rest of the 918 proteins remained insignificant (Fig. 6a). Among the upregulated proteins, ER-UPR-responsive proteins like Kar2, Pdi1, nucleotide exchange factor for Kar2, Sil1, were found along with many other differentially overexpressed proteins involved in the maintenance of proteostasis like Trx3, Grx3, Hsp42 etc. (Fig. 6c and Supplementary Table 3). When we compared the protein levels of Tm-treated *sse1*Δ cells with untreated *sse1*Δ cells, we found 75 proteins to be upregulated and 101 proteins to be differentially downregulated (Fig. 6a). Like WT cells, *sse1*Δ cells also showed differentially upregulated Kar2, Pdi1, Trx3, Grx3, Hsp42 upon Tm-treatment (Fig. 6d). Interestingly, a comparison of Tm-treated *sse1*Δ cells with respect to Tm-treated WT cells showed upregulation of 29 proteins and downregulation of 40 proteins. Among the upregulated proteins, both subunits of yeast nascent polypeptide chain associated complex (NAC), Egd1 (yeast ortholog of NACβ) and Egd2 (yeast ortholog of NACα) (Zhang *et al*. 2012) were present (Fig. 6e). The differential overexpression of NAC subunits in *sse1*Δ cells during ER stress indicates a cellular response to protect the newly synthesized proteins as NAC protects the nascent chains (NCs) from aggregation and misfolding upon emerging from ribosome exit tunnels (Rospert *et al*. 2002; Zhang *et al*. 2012). These data are in corroboration to unchanged level of ribosome-bound-chaperone, Ssb1, a well-known chaperone that binds the NCs upon emergence from ribosome-exit tunnels, in *sse1*Δ cells following Tm-induced ER stress (Supplementary Fig. 3c). In contrast, WT cells show a prominent reduction of Ssb1-bound to ribosomes following Tm-treatment (Supplementary Fig. 3c) in corroboration with the significantly decreased new protein translation upon Tm-treatment as described in the previous section. Thus, it is distinctly evident from multiple results that *sse1*Δ strain more efficiently continues protein translation during Tm-induced ER stress.

### Sse1 plays a crucial role in controlling ER-stress-induced cell division arrest and cell viability

**Fig. 7.**
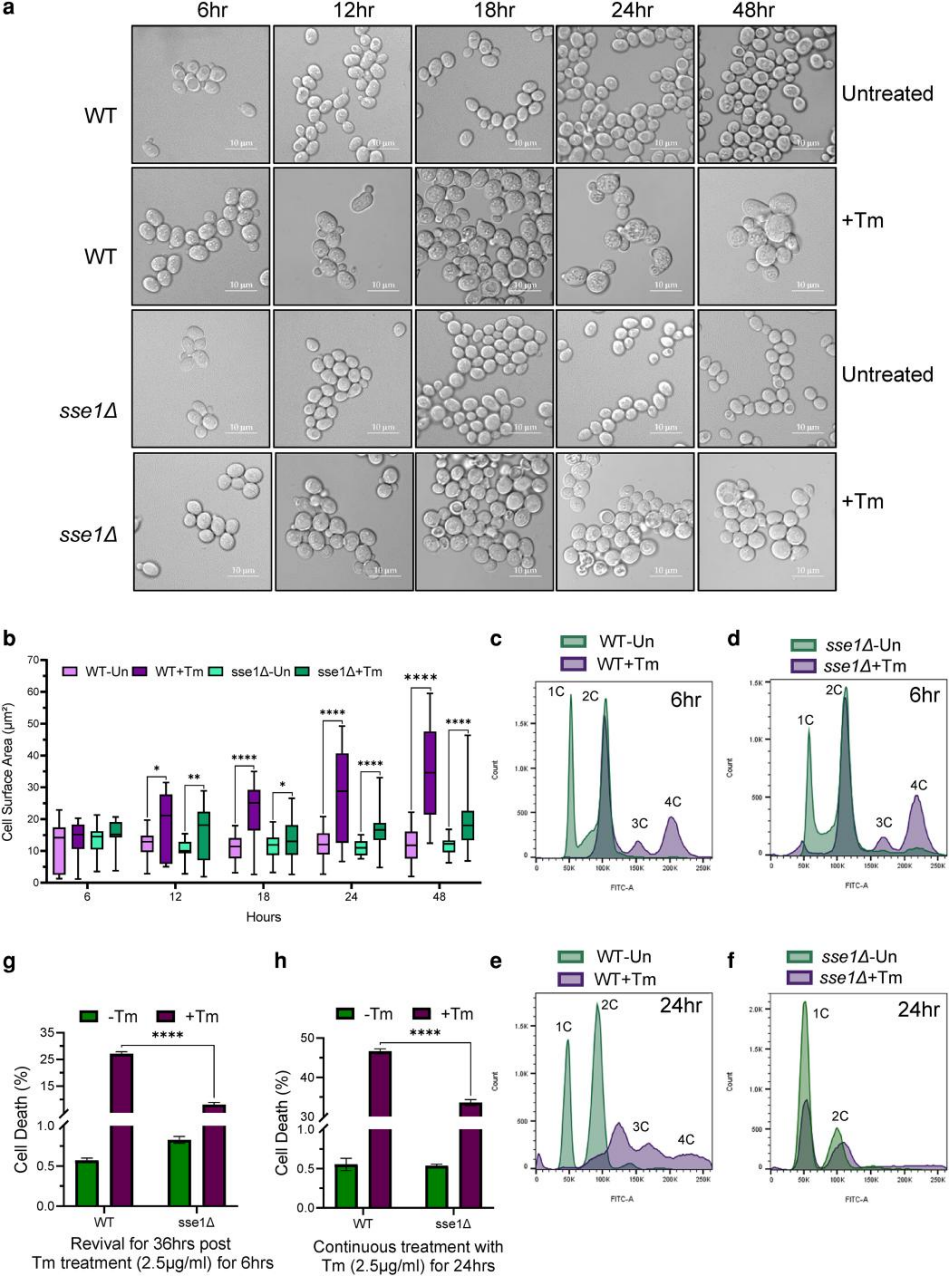
Sse1 controls tunicamycin-induced ER-stress-mediated cell division arrest and cell death of yeast. a) Images of WT and *sse1Δ* cells taken by confocal microscopy following treatment with Tm (2.5 µg/ml) along with the untreated control cells. Images were captured at 6, 12, 18, 24, and 48-h time points, respectively. b) The quantitation of the size (surface area in μm^2^) of the yeast cells from the previous panels, which are represented as a box and whiskers plot where the top whiskers represent the highest and the bottom whiskers represent the lowest individual cell surface area. The horizontal line within each box represents the median cell surface area. Statistical significance was calculated using unpaired T-tests and the pairwise comparison outputs were plotted in the graph (12 h: WT-Tm/WT + Tm, two-tailed *P* = 0.0144, *; *sse1*Δ-Tm/*sse1*Δ+Tm, two-tailed *P* = 0.0060, **; 18 hrs: WT-Tm/WT + Tm, two-tailed *P* < 0.0001, ****; *sse1*Δ-Tm/*sse1*Δ+Tm, two-tailed *P* = 0.0403, *; 24 hrs: WT-Tm/WT + Tm, two-tailed *P* < 0.0001, ****; *sse1*Δ-Tm/*sse1*Δ+Tm, two-tailed *P* < 0.0001, ****; 48 hrs: WT-Tm/WT + Tm, two-tailed *P* < 0.0001, ****; *sse1*Δ-Tm/*sse1*Δ+Tm, two-tailed *P* < 0.0001, ****). c–f) Cell cycle analysis was done for the WT and *sse1Δ* cells using the DNA binding fluorescent dye Sytox Green following treatment with Tm (2.5 µg/ml) along with untreated controls. The data were captured after 6 and 24 hrs of Tm (2.5 µg/ml) treatment. c) The overlaid histogram represents the pairwise comparison of WT-untreated/WT + Tm cell cycle pattern at 6 hrs post-Tm-treatment. d) The overlaid histogram represents the pairwise comparison of *sse1Δ*-untreated/*sse1Δ*+Tm cell cycle pattern at 6 hrs post-Tm-treatment. e) The overlaid histogram represents the pairwise comparison of WT-untreated/WT + Tm cell cycle pattern at 24 hrs post-Tm-treatment. f) The overlaid histogram represents the pairwise comparison of *sse1*-untreated/*sse1Δ*+Tm cell cycle pattern at 24 hrs post-Tm-treatment. g) Cell death percentage was analysed using propidium iodide staining through flow cytometry and was plotted as a bar plot (with whiskers representing SEM, *n* = 3) using the strains WT (BY4741) and *sse1*Δ (in BY4741 strain background) at the revival stage [after 36 hrs following optimal Tm (2.5 µg/ml) treatment for 6 hrs]. Statistical significance was calculated using unpaired T-tests and the significant pair was plotted in the graph (WT + Tm/*sse1*Δ+Tm, *P* < 0.0001, ****). h) A similar cell death percentage as shown in panel G was determined for WT (BY4741) and *sse1*Δ (in BY4741 strain background) after chronic ER stress of 24 hrs by Tm (2.5 µg/ml) treatment. Statistical significance was calculated using unpaired T-tests and the significant pair was plotted in the graph (WT + Tm/*sse1*Δ+Tm, *P* < 0.0001, ****).

As *sse1*Δ strain showed prominent growth fitness during long-standing Tm-stress and tunicamycin stress is known to cause cell division arrest in yeast (Babour *et al*. 2010; Niwa 2020), it was interesting to check the status of cell division of this deletion strain during Tm-induced ER stress. Furthermore, we found that mitotic cell cycle and cell cycle pathways to be significantly upregulated in Tm-treated *sse1*Δ cells by pathway enrichment analysis of the transcriptomics data, as described before (Fig. 5c). Thus, it prompted us to check the cellular morphology and cell cycle status of the Tm-untreated and treated WT and *sse1*Δ cells. To check the cellular morphology, we performed imaging of yeast cells by confocal microscopy at different time points following Tm-stress. WT cells showed significantly higher cell size (3 to 4 times) and granularity in the later time points of stress (most prominent from 18 h post-Tm-treatment) compared to *sse1*Δ cells (Fig. 7a and b). In the *sse1*Δ strain, the increase in cell size post-Tm-stress was also observed but to a much lesser extent compared to WT cells (Fig. 7a and b). These data indicated that there is a block in cell division in WT cells as a response to ER stress which is bypassed in the *sse1*Δ strain. To validate this finding, we did a cell cycle analysis with Sytox Green dye as described before (Rosebrock 2017). The untreated cells in both WT and *sse1*Δ strains showed cells possessing 1C and 2C DNA content (Fig. 7c and d). After 6 hrs of Tm-treatment, cell cycle analysis showed the appearance of populations with more DNA content (3C and 4C) in both the strains (Fig. 7c and d and Supplementary Fig. 4ai and ii) indicating cytokinesis arrest. Upon long-standing stress for 24 h of Tm-treatment, all the cells of the WT strain were populated in a higher DNA-containing population (3C, 4C) indicating a major block in cell division (Fig. 7e and Supplementary Fig. 4aiii). In sharp contrast, after 24 hrs of Tm-stress, *sse1*Δ strain showed a majority of the cells in the 1C and 2C population almost overlapping with the untreated condition (Fig. 7f and Supplementary Fig. 4aiv) indicating progression of cell division. These data are intriguing and indicate an important role of Sse1 in controlling cell division arrest during Tm-induced ER stress.

To test whether the escape of cell division arrest of *sse1*Δ strain during Tm-stress leads to alteration in cell viability following acute or chronic ER stress, we measured the cell viability using propidium iodide staining of yeast cells. In case of short-term Tm stress for 6 hrs followed by recovery for 36 hrs, it showed ∼27% cell death in WT cells (Fig. 7g). In contrast, cell death was significantly less (∼8%) in the case of *sse1*Δ cells strain (Fig. 7g). In case of uninterrupted chronic Tm-treatment, the percentage of cell death of *sse1*Δ cells was always significantly lower compared to WT cells (Fig. 7h and Supplementary Fig. 4b). These data together indicate that the Tm-resistance of the *sse1*Δ strain observed in growth assays is due to evasion of cell division arrest and significantly higher cell survival of the *sse1*Δ cells during Tm-induced ER stress. The role of Sse1 in controlling the cell division remains elusive at the moment. Altogether, we show an important role of Sse1 in preventing cell division and successful triggering of cell death pathways following Tm-induced ER stress.

## Discussion

The Hsp110 group of molecular chaperones is exclusively found in eukaryotes although it's Hsp70 partners are conserved across almost all kingdoms of life. As a representative member of Hsp110s, the cellular roles of yeast Hsp110, Sse1, have been extensively explored for more than the past two decades. Although Sse1's role as a potent NEF of cytosolic Hsp70s (Ssa and Ssb) is well documented, it's individual role in protein homeostasis beyond cochaperone activity, if any, is not much explored. In this work, we reveal a yet unexplored role of Sse1 in modulating the ER-unfolded protein response during tunicamycin-induced ER stress. We show that Sse1 is required for an optimum cellular response during overwhelming ER stress caused by tunicamycin (Tm), an N-linked glycosylation inhibitor. Our data reveal that in the absence of Sse1, cells acquire an unusual resistance to ER stress. Importantly, this Tm-resistance of *sse1Δ* strain is critically dependent on the successful induction of Ire1-Hac1 signaling mediated ER-UPR. In lower concentrations of Tm, *sse1Δ* does not show any resistance to the stressor due to insufficient mounting of ER-UPR. To explain the Tm-resistance, we initially hypothesized a multitude of possibilities like; (1) high basal heat shock response (HSR) of *sse1Δ* strain and (2) decreased load of newly synthesized ER proteins due to the absence of CLIPS function of Sse1 which could be beneficial during ER stress. In contrast, recent literature has shown that the efficiency of ER-reflux of proteins is reduced in the absence of Sse1 during Tm-stress (Igbaria *et al*. 2019), which may increase the load of aberrantly folded proteins inside ER and compromise the ER protein homeostasis leading to higher basal ER-UPR in *sse1*Δ cells. In that scenario, the cellular fitness of yeast in the absence of Sse1 is counter-intuitive.

**Fig. 8.**
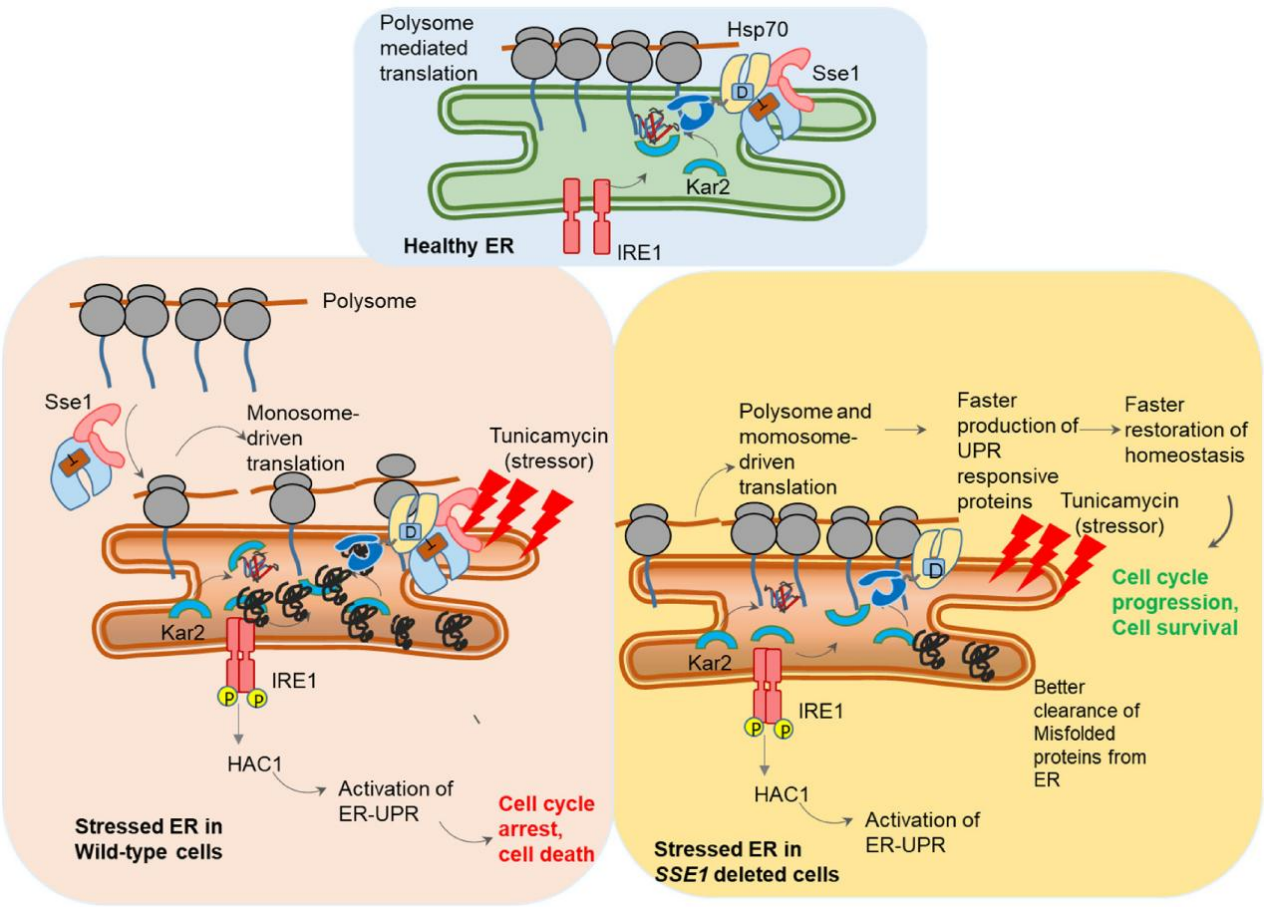
A schematic summary of the role of Sse1 during ER stress. A schematic model summarizing the role of Sse1 during ER stress. The upper box represents the physiological condition when there is no stress to ER. The lower left box summarizes the condition of WT cells during Tm-mediated ER stress where polysomes are majorly reorganized into monosomes, and in this process Sse1 plays an important role. There is UPR induction by activation of Ire1-Hac1 pathway, which restores homeostasis up to a tolerable level of stress. The lower right box summarizes the condition of *sse1*Δ cells during ER stress with tunicamycin. Polysome to monosome conversion is inefficient in absence of Sse1 which leads to faster production of UPR-responsive proteins which in turn restores the homeostasis faster. Additionally, the ER stress induced cell cycle-arrest is evaded in *sse1*Δ cells leading to fitness advantage during tunicamycin stress and more cell viability.

To find out the most probable explanation of the Tm-resistance of the *sse1Δ* strain, we explored further and ruled out the contribution of high basal HSR as other strains possessing high basal HSR or constitutive activation of HSR do not show similar Tm-resistance. Interestingly, deletion strains of two other CLIPS, *JJJ1* and *GIM2* apart from *SSE1* show the same Tm-resistance phenotype indicating the importance of the absence of individual CLIPS function as one of the common factors to gain Tm-resistance. This result prompted us to explore the role of Sse1 in modulating the protein translation status during Tm-induced ER stress. We found that Sse1 plays a crucial role in the stress-induced reorganization of the majority of translating ribosomes from polysomes to monosomes. In the absence of Sse1, this ribosomal reorganization is inefficient leading to a less prominent reduction in polysome fraction and such ribosomal status in the *sse1*Δ cells leads to continued protein translation during long-standing Tm-induced ER stress, in complete contrast to ceased protein translation in WT cells. Our initial hypothesis was that the continuation of protein translation leads to a more efficient synthesis of ER-UPR-induced genes leading to better ER-stress management by *sse1Δ* strain. To check this, when we monitored the ER-UPR kinetics, we found the kinetics of UPR is prominently different from WT cells. *sse1Δ* strain shows faster activation as well as faster reversal from the UPR-activated state. This result indeed shows that *sse1Δ* strain can activate the ER-UPR faster and restore the homeostasis quicker than WT leading to the fitness advantage during Tm-stress (summarized schematically in Fig. 8).

It was previously shown that Tm-induced ER stress is known to cause cytokinesis arrest in yeast (Babour *et al*. 2010; Niwa 2020). As *sse1Δ* strain continued to grow in the presence of Tm-stress, it prompted us to check the cell division status of this strain following ER stress. Importantly, a cell cycle analysis revealed that the cytokinesis arrest, observed in WT cells following long-standing Tm-induced ER stress, is absent in *sse1Δ* strain. The progression of cell division in the *sse1Δ* strain can explain the significantly higher rate of synthesis of new proteins as detected by AHA incorporation by Click-IT reaction following ER stress in contrast to WT cells. Importantly, the reversal to the basal state from the UPR-activated state (post 24 hrs of Tm-treatment) coincided with the reversal of the 3C/4C population to 1C/2C states in cell cycle analysis indicating progression of the cell cycle in the *sse1Δ* strain. Thus, a quicker reversal from the UPR-activated state of the *sse1Δ* strain can be due to cell division and new cell formation. These data nicely corroborated with the unique enrichment of mitotic cell cycle and cell cycle pathnways as differentially upregulated pathways exclusively in the transcriptome of Tm-treated *sse1Δ* cells, as described before (Fig. 5c). This is important to mention here that we observed differential overexpression of the MAP kinase Slt2 by quantitative mass spectrometry which is critical for yeast cell wall integrity (Gonzalez-Rubio *et al*. 2022; Sanchez-Adria *et al*. 2022; Sanz *et al*. 2022) upon Tm-treatment in both WT and *sse1*Δ cells (Fig. 6c and d). Importantly, Slt2 was also shown to prevent cell division during Tm-induced ER stress by septin ring mislocalization and cytokinesis block (Babour *et al*. 2010; Niwa 2020). Additionally, a previous study described that Slt2 interacts with Sse1 and is partially dependent on Sse1 for its cellular activities although absence of Sse1 does not alter the Slt2 protein quantity or its phosphorylation status (Shaner *et al*. 2008). Thus, despite overexpression, Slt2 can be inefficient in septin ring mislocalization and cytokinesis block in *sse1*Δ strain leading to cell division progression.

We assume that continued protein translation of the *sse1*Δ strain leading to quicker ER-UPR induction and restoration of homeostasis leads to escape from cell division arrest and fitness during Tm-stress (summarized in Fig. 8).

Taken together, our data show the importance of cytosolic chaperone Sse1 in maintaining ER proteostasis in physiological conditions and during overwhelming ER stress by stressors like tunicamycin. We could not establish the molecular mechanism of modulatory role of Sse1 on ER-UPR. Although we convincingly show the genetic interaction of *SSE1* with ER-UPR sensors like *IRE1* or *HAC1*, the molecular basis of such genetic interaction remains elusive and requires further investigation. Furthermore, we show interesting genetic interactions with other CLIPS chaperones like Jjj1 and Gim2 in the absence as well as in the presence of Tm-induced ER stress. The molecular mechanism of Sse1's role in controlling cell division arrest during ER stress and how the process is escaped in the absence of a functional chaperone remains to be explored in detail in the future. It is important to note that molecular chaperones like Sse1 are thus important in controlling the passage of stress-damaged ER to the progeny by controlling the cell division and helping in triggering cell death in case of overwhelming ER stress. Furthermore, if the phenomenon of ER-stress resistance due to inactivity of Hsp110 chaperones remains conserved in higher eukaryotes, any mutation leading to nonfunctionality of Hsp110s may have wide-ranging implications in pathologies where ER-UPR is elicited.

## Supplementary Material

jkae075_Supplementary_Data

## Data Availability

The mass spectrometry proteomics data have been deposited to the ProteomeXchange Consortium via the PRIDE partner repository (Perez-Riverol *et al*. 2022) with the dataset identifier PXD045382. The transcriptomics data have been deposited in NCBI with the bioproject accession number PRJNA1026743. Yeast strains and plasmids are available upon request. Supplemental material available at G3 online.
