# Supplementary material for: Sse1, Hsp110 chaperone of yeast, controls the cellular fate during endoplasmic reticulum stress
Source: G3 (Bethesda). 2024 Apr 5;14(6):jkae075. doi: 10.1093/g3journal/jkae075 (PMC11152076; doi:10.1093/g3journal/jkae075)
Supplement: jkae075_Supplementary_Data [file jkae075_supplementary_data.zip › Supplemental_Material_Text_G3-2024-404855.docx]

**Supplementary Information**

**Sse1, Hsp110 chaperone of yeast, controls the cellular fate during Endoplasmic Reticulum stress**

**Mainak Pratim Jha^1^, Vignesh Kumar^2,3^, , Asmita Ghosh^2,3^ and Koyeli Mapa^1*^**

1. Protein Homeostasis Laboratory, Department of Life Sciences, School of Natural Sciences, Shiv Nadar Institute of Eminence, Delhi-NCR, Greater Noida, Gautam Buddha Nagar, Uttar Pradesh 201314, India.

2. Chemical and Systems Biology Unit, CSIR–Institute of Genomics and Integrative Biology, New Delhi 110025, India.

3. Academy of Scientific and Innovative Research (AcSIR), Ghaziabad 201002, India.

* Correspondence: [koyeli.mapa@snu.edu.in](mailto:koyeli.mapa@snu.edu.in)

**Table of content**

Supplementary Figure legends (Figure S1-S5)

Supplementary Methods

Table S1 (list of plasmids and strains used in the study)

Table S2 (transcriptomics raw data, as an excel sheet)

Table S3 ( quantitative proteomics raw data, as an excel sheet)

Table S4 (list of transcription factors in the enriched pathways as described in the Figures 5B and 5C, as an excel sheet)

**Supplementary Figure legends**

**Figure S1**

**A.**  Yeast growth assay by serial drop dilutions using the strains WT (BY4741), *sse1Δ*, and *sse2Δ* in YPAD plates at permissive temperature (30°C) and in presence of the following proteotoxic stresses: oxidative stress [4mM hydrogen peroxide(H_2_O_2_)], nutrient starvation (0.1% glucose), Hsp90 inhibition by geldanamycin (50mM), , reducing stress (by DTT), DNA damaging agent and mitochondrial stressor (Ethidium Bromide, 1µg/ml), general proteotoxic stress by proline analogue L-Azetidine-2-Carboxylic Acid (AZC, 2.5 µM), and mitochondrial oxidative phosphorylation inhibitor and protonophore, CCCP, (20µM Carbonyl Cyanide m-Chloro-Phenyl hydrazone). The 30°C panel without any stressor as shown in Figure 1Ai left panel, has been shown again here for easy comparison of phenotypes without and with stressors. All treatments with stressors were done at 30°C. The triangle above each panel indicates the increasing dilutions. The time mentioned in hours represents the time of incubation before taking the image of the plates. Aii. The growth curves of WT, *sse1Δ*, and *sse2Δ* done in liquid media (YPD) at 30°C are shown. **B.** (**Bi)** Drop dilution assay using the WT, *sse1*Δ, and *sse2*Δ strains in YPD plates at permissive temperature (30°C) and in presence of protein translation blocker Cycloheximide (40 ng/ml). (**Bii)** Growth curves of three strains used in panel (Bi) in liquid media in presence of cycloheximide (40 ng/ml) in YPD are shown as stated earlier in (Aii). **C.** Yeast growth assay by serial drop dilutions using the strains wild-type (BY4741), *sse1Δ* transformed with either the empty plasmid vectors (EV or vector control) or the plasmids expressing the wild type Sse1 protein or its mutant versions at endogenous level (from pRS315 plasmid. *sse1Δ* strain was transformed with the ATP hydrolysis mutant of Sse1 (Sse1-K69Q), and the ATP binding mutants (Sse1-G205D, and Sse1-G233D) expressed under Sse1 native promoter. The drop dilution assay was performed using the strains wild-type (BY4741) + pRS315 (Empty Vector, EV), *sse1Δ* + pRS315 (Empty Vector, EV), *sse1Δ* + pRS315 – Sse1, *sse1Δ* + pRS315 – Sse1-K69Q, and *sse1Δ* + pRS315 – Sse1-G205D, *sse1Δ* + pRS315 – Sse1-G233D, in SD-Leu (synthetic media with Dextrose without leucine) agar plates in permissive temperature (30°C), and in presence of the optimum tunicamycin (2.5 µg/ml) concentrations sufficient to mount ER-UPR. **D.** Drop dilution assay using the same strains as shown in panels A, in presence of the ER stressor Tm in sub-optimal (2.5 ng/ml and 5.0 ng/ml) concentrations. **E. (Ei)** Western blot showing no prominent change in Kar2 levels in response to suboptimal (2.5 ng/ml) concentration of Tunicamycin indicating no activation of ER-UPR at this concentration of Tm. GAPDH was used as the loading control. **(Eii)** The bands were quantified by densitometry and were plotted as a bar plot with whisks representing SEM in the right-side panel. Statistical significance was calculated using unpaired T-tests and none of the pairwise comparisons was found to be significant. **F**. The presence of different *HAC1* mRNA variants after suboptimal tunicamycin (2.5 ng/ml) treated condition was checked as described in Figure 1C. After Tm-treatment, yeast cells were harvested, RNAs were extracted and from that cDNAs were made. Then from the cDNAs by PCR amplifications with the help of specific primers, we checked the spliced and unspliced forms. The band intensities were measured by densitometry and were plotted as a bar plot as shown in the below panel (n=3). Statistical significance was calculated using unpaired T-tests and the significant pairs were plotted in the graph (Hac1s-Wt-Tm/Hac1s-sse1Δ-Tm, p=0.0466, *; and Hac1s-Wt+Tm/Hac1s-sse1Δ+Tm, p=0.0001, ****). **G.** Comparison of the level of glycoproteins present in wild-type (BY4741) and *sse1Δ* cells under the physiological conditions. To detect this, we grew the wild-type (BY4741) and *sse1Δ* cells as a primary culture in YEPD (1% Yeast extract, 2% Peptone and 2% Dextrose) overnight to get a saturated culture. The next day, the secondary cultures were inoculated at 0.1 OD_600_ and then allowed to grow till it reaches the mid-log phase. Then the cells were harvested and the total proteins were extracted and subjected to binding to Concanavalin A agarose resins that bind all the glycoproteins. The resins were washed to remove the non-specifically bound proteins and bound glycoproteins were loaded in SDS-PAGE (left panel) and visualised through stain free UV-vis visualization system. Total cell lysates were also loaded (right panel) to visualize equal loading.

**Figure S2**

**A.** Yeast growth assay by serial drop dilutions using the strains WT (BY4741), *sse1Δ*, and *sse2Δ* in YPAD plates at permissive temperature (30°C) and in presence of different concentrations (0.1 µg/ml, 0.2 µg/ml, 0.5 µg/ml, 1.0 µg/ml, 1. µg/ml, 2.0 µg/ml and 6.0 µg/ml,) of tunicamycin was done. Tm-resistance of *sse1Δ* is observed at all concentrations of Tm more than or equal to 1.0 µg/ml. **B**. Drop dilution assay using the strains WT, and single deletion strains *sse1Δ*, *hac1Δ*, *ire1Δ*, and double deletion strains, *hac1Δ-sse1Δ* and *ire1Δ-sse1Δ* in YPAD plates at sub-optimal concentrations of Tm (2.5 ng/ml and 5.0 ng/ml). **C**. Yeast growth assay by serial drop dilutions using the strains wild-type (BY4741), *sse1Δ* along with the double deletion mutants with *sse1Δ* of the CLIPS strains that showed growth fitness with Tm-induced ER stress. The drop dilution assay was performed using the strains wild-type (BY4741), *sse1Δ*, *jjj1Δ*, *gim2Δ*, , *jjj1Δ-sse1Δ*, and *gim2Δ-sse1Δ*, in YPAD plates in permissive temperature (30°C), heat stress condition (37°C), and in presence of the optimal tunicamycin (2.5 µg/ml) concentrations. **D**. Here we wanted to verify the phenotypes of the two different background strains of wild-type yeast and the respective knockout strains made in these backgrounds. The drop dilution assay was performed using the strains wild-type (BY4741), wild-type (*yMJ003*), *sse1Δ* (BY4741), *sse1Δ* (*yMJ003*), *sse2Δ* (BY4741), and *sse2Δ* (*yMJ003*) in YPAD plates in permissive temperature (30°C), and heat stress condition (37°C).

**Figure S3**

**A**. The rate of translation of WT, and *sse1Δ* strains in untreated and Tm (2.5 µg/ml) treated conditions were analyzed using the CLICK-IT chemistry reaction using L-Azido-homoalanine and Alexa-Fluor 488 alkyne dye as described in figure 3B. The incorporated fluorescence in newly synthesized proteins was measured in each sample by flow cytometry and was plotted as a bar plot with whisks representing SEM. The panel shows the incorporated fluorescence following 48 hours of Tm-stress in comparison to untreated cells of WT and *sse1Δ* strains. Statistical significance was calculated using unpaired T-tests and the pairwise comparison outputs were plotted in the graph. (WT-Tm/WT+Tm, two-tailed p=0.0003, ***; *sse1*Δ-Tm/*sse1*Δ+Tm, two-tailed p=0.0008, *; WT+Tm/*sse1*Δ+Tm, two-tailed p<0.0001). **B**. The kinetic change in the number of cells of the two population, P1 & P2, marked in the main figure 3D are represented as stacked columns over time for the same set of WT, *sse1*Δ and *sse2*Δ cells. The whisks over each time point value represents SEM. **C.** Western blot showing the Ssb1 (left upper panel) and Sse1 (right upper panel) **.** (level in ribosome-bound fractions of the wild-type, *sse1*Δ strains in untreated and Tm (2.5 µg/ml) treated conditions. Amido black stained PVDF membrane after western blot transfer is shown as the loading control. Ssb1 quantification is shown in left bottom panel and Sse1 quantification is shown in right bottom panel. The bands were quantified by densitometry and were plotted as bar plots with whisks representing SEM (n=3). Statistical significance was calculated using unpaired T-tests and the significant pairs were plotted in the graph (For bottom left panel G: Wt-Tm/Wt+Tm, p=0.0067, **; and sse1Δ-Tm/*sse1*Δ+Tm, p=0.2119, ns; For bottom right panel H: Wt-Tm/Wt+Tm, p=0.0079, **). The above pairwise comparisons, except the pair *sse1*Δ-Tm/*sse1*Δ+Tm for panel G, are significant even after Bonferroni correction.

**Figure S4**

**A**. The overlaid histograms of cell cycle analysis shown in Figure 7C-F are shown as 3D- histograms where they represent the following pairwise comparisons: **(i)** WT-untreated/WT+Tm cell cycle pattern at 6hours post Tm treatment, **(ii)** *sse1Δ*-untreated/*sse1Δ*+Tm cell cycle pattern at 6 hours post Tm treatment, **(iii)** WT-untreated/WT+Tm cell cycle pattern at 24 hours post Tm treatment, and **(iv)** *sse1Δ*-untreated/*sse1Δ*+Tm cell cycle pattern at 24 hours post Tm treatment.. **B**. Cell death percentage was analysed by propidium iodide (PI) staining by flow cytometry and was plotted as a bar plot with whisks representing SEM using the strains wild-type (BY4741) and *sse1*Δ and *sse2*Δ strains (in BY4741 strain background). The measurements were done at different time points ( 48, 72, 96 and 120 hours) after continuous treatment with optimum tunicamycin (2.5 µg/ml) concentration. Finally, the cell death percentages were plotted for the untreated and treated conditions for the above-mentioned strains at the specified time points. Statistical significance was calculated using unpaired T-tests along with setting the α at 95% and the significant pairs were plotted in the graph (Wt+Tm/*sse1*Δ+Tm for 48 Hr, p=0.0001, ****; Wt+Tm/*sse1*Δ+Tm for 72 Hr, p=0.1395, ns, Wt+Tm/*sse1*Δ+Tm for 96 Hr, p=0.0066, **; and Wt+Tm/*sse1*Δ+Tm for 120 Hr, p=0.0071, **). The above p-values, except the second pair Wt+Tm/*sse1*Δ+Tm for 72 Hr, are significant even after Bonferroni correction.

**Figure S5**

**A.** The spots of the strains from Figure 2-A panel drop dilution assays were quantified using densitometry and were plotted as a bar plot with whisks representing SEM as shown in the right panel (n=3). Statistical significance was calculated using unpaired T-tests and the significant pairs were plotted in the graph (all comparisons had p<0.0001, and they were significant even after Bonferroni Correction). **B**. The spots of the strains from Figure 2 B and C panel drop dilution assays were quantified using densitometry and were plotted as a bar plot with whisks representing SEM as shown in the right panel (n=3). Statistical significance was calculated using unpaired T-tests and the significant pairs were plotted in the graph (all comparisons were significant even after Bonferroni Correction). **C**. The spots of the strains from Figure 2-D panel drop dilution assays were quantified using densitometry and were plotted as a bar plot with whisks representing SEM as shown in the right panel (n=3). Here it is clearly evident that the sse1∆ strain is significantly sick under permissive temperature and significantly fit under Tm-induced ER-UPR conditions when compared to any of the other mutants, so only the bars representing the sse1∆ strain are marked with a star in the figure. **D**. The spots of the strains from Figure 2 E panel drop dilution assays were quantified using densitometry and were plotted as a bar plot with whisks representing SEM as shown in the right panel (n=3). Statistical significance was calculated using unpaired T-tests and the significant pairs were plotted in the graph (all comparisons were significant even after Bonferroni Correction). **E**. The spots of the strains from Figure 2-D panel drop dilution assays were quantified using densitometry and were plotted as a bar plot with whisks representing SEM as shown in the right panel (n=3). Here it is clearly evident that along with the sse1∆ strain, there are these three strains, jjj1∆, gim2∆, and cct8∆, which are significantly fit under Tm-induced ER-UPR conditions when compared to any of the other mutants, so only the bars representing the fit strains are marked with a star in the figure.

**Supplementary Methods**

**Sample preparation and Quantitative Mass Spectrometry**

Specific yeast strains are grown according to the experimental setup and the desired treatments were given accordingly, following which the cells were harvested at 8000 RPM and washed with sterile MQ water once. After that, the cells were resuspended in 0.1 M sodium hydroxide and kept at room temperature for 30 minutes. The cells were then centrifuged at 4000 RPM and resuspended in lysis buffer (40mM Tris pH 6.8, 10% Glycerol, 2% SDS), usually 200-300µl, 300mg acid-washed glass beads were added and then bead beating was done followed by incubation at 95°C for 10 minutes. Then the samples were centrifuged at 12500 RPM for 15 minutes and the supernatants were transferred to a fresh tube and the protein concentrations were estimated by the BCA protein estimation method. All the samples were normalised according to their concentrations and proceeded for acetone precipitation. The acetone to be used was kept in a glass container at -20°C for 1 hour. Approximately 50µg of protein were taken for each sample in a separate tube and acetone was added four times the protein volume (approximately 600µl were added to each tube). The tubes were then vortexed briefly and kept at -20°C for overnight. After that, the samples were centrifuged at 10000 RCF for 15 minutes at 4°C. Following this, the supernatants were discarded and the pellets were air dried till they became transparent. Then the pellets were resuspended in the basic buffer (100mM Tris pH-8, 8M Urea), usually 40-50µl, and then protein concentration estimation was performed through the BCA method. To proceed with Mass Spectrometry, now the proteins were digested with trypsin, and 20µg of protein were taken from each sample for a total 25µl reaction volume where 2µl of dithiothreitol (DTT, 25mM) was added to reduce the disulphide bonds and incubated at 60°C for 30 minutes. Then 1 µl of iodoacetamide (IAA, 50mM) were added to each tube and incubated in a dark environment at room temperature for 20 minutes. Now the urea concentration in the buffer is diluted to 1 M from 8 M. After that trypsin (resuspended in 100mM Tris pH-8, 1 M Urea buffer at a 1µg/ml concentration) was added to the vials following a 1:10 ratio for the enzyme to protein (for 20µg protein 2µg trypsin) and incubated at 37°C on a thermomixer for 16-18 hours. After the incubation is over the enzyme was inactivated by adding formic acid (0.1% v/v to the final enzyme concentration) to each tube, following that all the digested peptides were dried using a vacuum and stored at -80°C until further use. After that, the peptides were desalted using the C18 Ziptips (Millipore) as per the manufacturer’s protocol, following that the samples were lyophilized and loaded onto the LC-MS setup of the ABSciex 6600 Hybrid mass spectrometer instrument that ran on high-sensitivity SWATH mode. After the mass spectrometer run, the protein peak identification and label-free quantitation were done in the proprietary software suite that came along with the mass spectrometer. After the expression values were generated for all the identified proteins, statistical significance was calculated. The fold change analysis was done on the raw expression values and then Log_2_ transformed, whereas t-test significance was calculated on the Log_2_ transformed expression values and then the calculated p-value was then readjusted by applying antilog with base 10. Finally, all the volcano plots were plotted with the ‘Log_2_ – Fold Change’ on the X axis and the ‘-Log_10_(P)’ value on the Y axis.

**Confocal Microscopy of Yeast**

To assess the overall morphology of yeast cells under normal and treatment conditions we visualised them under the Nikon A1R MP^+^ Multiphoton Confocal Microscope. Specific yeast cells were grown according to the standard primary and secondary culture protocol as per the experimental setup. After the treatments are done aliquots of cells were collected, washed with 1X PBS and resuspended in a small volume of 1X PBS. Then a small volume of the resuspended cells (usually 5-10µl) was placed on top of agarose pads (1% agarose in 1X PBS) prepared previously on glass slides and a cover slip was placed on top of that and visualised under the microscope. The quantitation associated with the confocal microscopic images was done in the proprietary Nikon NIS-Elements AR Analysis 5.20.02 software and represented through suitable graphical forms.

**Propidium Iodide (PI) Staining and Flow Cytometry**

We used the PI staining for measuring the cell death, respective yeast strains were grown as required by each experimental setup. After the specific treatments were done a maximum of 1 OD_600_ (approximately 10^6^) cells were harvested at 8000 RPM for 5 minutes. A control strain cell pellets were collected separately, re-suspended in 200µl of 1X PBS and kept at 95°C for 15 minutes. Cells were harvested at 8000 RPM for 5 minutes, and these cells served as the positive control for the PI staining procedure. Re-suspend all the test cell pellets in 200µl of 1X PBS and 1µl of PI stain (1mg/ml) were added to all the re-suspended cell suspensions, only leaving the negative control strains. All the samples were incubated for 10 minutes at room temperature in dark conditions. Then all the samples were harvested at 8000 RPM for 5 minutes, the supernatant was discarded leaving the cell pellet. All the samples including positive and negative control strains were re-suspended in 300µl of 1X PBS and proceeded for flow cytometry analysis. We used the CytoFlex-S flow cytometer (Beckman Coulter), and the PI stain was detected using the PE channel. In the case of each strain, 50,000 single cells were recorded; live and dead cell populations were detected using the negative and positive control gating respectively and the settings were applied to all the test samples and evaluated accordingly.

**Cell Cycle analysis by Flow Cytometry**

We used cell cycle analysis to assess the cell cycle stages within which the cells are stuck in response to chronic ER stress elicited by tunicamycin treatment the in WT and *sse1*Δ yeast strains. These yeast strains were reinoculated in the secondary cultures at 0.1 OD_600_ from the overnight grown primary cultures and were allowed to grow till 0.4-0.6 OD_600_ in YPD media. After that cells were treated with optimal concentrations of tunicamycin (2.5 µg/ml) and cells were taken out post 6hrs, 24 hrs and 48 hrs of Tm treatment. At each time point, cells were centrifuged at 8000 RPM for 5 minutes, washed with sterile water and then centrifuged at 8000 RPM for 5 minutes and after that the supernatant was discarded. Then the cells were resuspended in 53% (v/v) absolute ethanol in 1X PBS and kept in a shaker at 14.8°C for 40 minutes at 200 RPM. Next, the cells were harvested at 8000 RPM and the supernatant was removed. The cells were then resuspended in 500µl RNA digestion and DNA staining buffer [50mM Sodium Citrate (pH 7.2 adjusted with Citric Acid), RNase A 20µg/ml, and Sytox Green (Invitrogen – S7020) 2.5µM] and incubated for 1 hour at 37°C in the dark with shaking at 200 RPM. After that 10µl of 20mg/ml of Proteinase K was added to the buffer, mixed thoroughly, and incubated in the dark at 55°C for 1 hour with shaking at 200 RPM. After the incubation was done, cells were resuspended in the buffer and were kept at 4°C until ready for flow cytometric data acquisition. We used BD LSR 2 Flow Cytometer for capturing this set of data with the following gating settings as described before [4], 1. First, through a scatter plot of FSC-area (X-axis) vs SSC-area (Y-axis), we gated the total area only leaving the debris part. 2. Next through a scatter plot of FSC-area (X-axis) vs FSC-width (Y-axis) we gated the linear population area leaving all the remaining parts. 3. Next, through a scatter plot of SSC-area (X-axis) vs SSC-width (Y-axis), we gated the linear population area leaving all the remaining parts. 4. Next through a scatter plot of Sytox Green-area (X-axis) vs Sytox Green-width (Y-axis), we gated the linear population area leaving all the remaining parts. 5. Finally, through a histogram of Sytox Green-area (X-axis) vs count we visualized the different stages of cells with individual peaks representing unique cell cycle stages. All the above plots were kept at a linear scale. The fluorescence was detected using the FITC channel and in the case of each strain and each data point For each sample measurement, 50,000 individual cells were recorded. After the acquisition, the data were processed and represented as shown in the results section.

**Purification of Glycoproteins**

We used Concanavalin A (Con A) Agarose (from G Biosciences with Cat No. 786-216) to purify the glycoproteins from the total cellular lysate to assess the changes occurred in the glycoprotein pool of the yeast strains in response to our treatment condition. Respective yeast strains were inoculated as primary cultures and allowed to grow overnight to yield a saturated culture the next morning. Then from that, each of the strains was reinoculated as a secondary culture at 0.1 OD_600_ and was allowed to grow till 0.4-0.6 OD_600_. Then according to the experimental protocol desired treatments were given, and in this case, the Wt. and sse1∆ cells were treated with 2.5µg/ml of tunicamycin for 6 hours and then the cells were harvested at 8000 RPM for 5 minutes, the supernatants were discarded, and the cell pellets were processed for total cellular protein extraction as described earlier in this methodology’s Western Blot section. After the extraction of the total cellular protein, their concentrations were normalised and proceeded for glycoprotein purification. For each sample 40µl of Concanavalin A (Con A) Agarose resins (dissolved in absolute ethanol in 1:1 dilution) were taken for 300µg of protein in a separate Eppendorf tube and centrifuged at 4000 RPM for 2 minutes. The supernatants were discarded, and the resins were washed with 200µl (that is 5 column volume) of Equilibration buffer (1 M Sodium Chloride, 5 mM Magnesium Chloride, 5 mM Manganese Chloride, 5 mM Calcium Chloride and the pH was adjusted to 7.4) to assure that Mn^2+^ and Ca^2+^ ions are present along with the resins for binding the glycoproteins efficiently. Then the resins were centrifuged at 4000 RPM for 2 minutes. The supernatants were discarded, and the resins were washed with 120µl (that is 3 column volume) of Binding buffer (20 mM Tris pH 7.4, and 0.5 M Sodium Chloride for limiting nonspecific binding). Then the resins were centrifuged at 4000 RPM for 2 minutes and the supernatants were discarded. After that, the resins were mixed with 300µg of total cell lysate protein and incubated in a spiral rocker with the rotation set at 10 units for 30 minutes. After that 200µl (that is 5 column volume) of Binding buffer was added to each resin-protein mixture and incubated in a spiral rocker with the rotation set at 10 units for 30 minutes. Then the resins were centrifuged at 4000 RPM for 2 minutes and the supernatants were discarded leaving the resins bound with glycoproteins. Then each of the resin-protein sample mixtures was resuspended in 48µl of protein storage buffer (10mM Tris-HCl, 1mM EDTA, 150mM KCl, 1mM PMSF) and then mixed with 12µl of 5X SDS loading dye (10% Glycerol, 2% SDS, 5% β-ME, 20 mg Bromophenol Blue, 62.5 mM Tris pH 6.8). After that, the whole mixtures were incubated at 95ºC for 10-15 minutes with intermittent vortexing. Then the samples were centrifuged at 14000 RPM for 2 minutes and 20µl of the supernatant was loaded from each sample to each of the lanes of an SDS-PAGE gel and visualized through UV-Vis stain-free imaging system.
