# Supplementary material for: Sse1, Hsp110 chaperone of yeast, controls the cellular fate during endoplasmic reticulum stress
Source: G3 (Bethesda). 2024 Apr 5;14(6):jkae075. doi: 10.1093/g3journal/jkae075 (PMC11152076; doi:10.1093/g3journal/jkae075)
Supplement: jkae075_Supplementary_Data [file jkae075_supplementary_data.zip › Table_S1_G3-2024-404855.docx]

**Table S1 (list of plasmids and strains used in the study)**

**List of the plasmids used for this study:**

| **S.No** | **Plasmid** | **Alias** | **Description** | **Reference/ Source** |
| --- | --- | --- | --- | --- |
| **1** | **pRS315** | pRS315-LEU-CEN | **Empty vector, centromeric** | Sikorski et al., 1989 |
| **2** | **pJV340** | pPS189-LEU-2μ | **Empty vector, overexpression** | Kumar et al., 2020 |
| **3** | **pRS315-SSE1** | pRS315-SSE1-LEU-CEN | **SSE1 physiological expression** | Kumar et al., 2020 |
| **4** | **pJV340-SSE1** | pPS189-SSE1-LEU-2μ | **SSE1 overexpression** | Kumar et al., 2020 |
| **5** | **pJV340-FES1** | pPS189-FES1-LEU-2μ | **FES1 overexpression** | Kumar et al., 2020 |
| **6** | **pRS315-SSE1-K69Q** | pRS315-SSE1-K69Q-LEU-CEN | **SSE1-K69Q enodogenous expression** | Kumar et al., 2020 |
| **7** | **pRS315-SSE1-G205D** | pRS315-SSE1-G205D-LEU-CEN | **SSE1-G205D enodogenous expression** | Kumar et al., 2020 |
| **8** | **pRS315-SSE1-G233D** | pRS315-SSE1-G233D-LEU-CEN | **SSE1-G233D enodogenous expression** | Kumar et al., 2020 |
| **9** | **pRS316** | pRS316-URA-CEN | **Empty vector, centromeric** | Sikorski et al., 1989 |
| **10** | **BG1805-SSE2** | BG1805-SSE2-URA-2μ | **SSE2 overexpression** | Grayhack / Thermo |
| **11** | **pRS316-HSF1** | pRS316-HSF1-URA-CEN | **HSF1 enodogenous expression** | Gift from Dr Kausik Chakraborty’s lab at IGIB, Delhi |
| **12** | **pRS316-HSF1-R206S** | pRS316-HSF1-R206S-URA-CEN | **HSF1-R206S enodogenous expression** | Gift from Dr Kausik Chakraborty’s lab at IGIB, Delhi |

**List of the yeast strains used for this study:**

| **S.No** | **Strain Name** | **Genotype** | **Common name** | **Parent strain** | **Reference/ Source** |
| --- | --- | --- | --- | --- | --- |
| **1** | **BY4741** | MATa *his3Δ1 leu2Δ0 met15Δ0 ura3Δ0* | **WT** | S288C | S288C/ EUROSCARF |
| **2** | ***sse1∆*** | MATa *his3Δ1 leu2Δ0 met15Δ0 ura3Δ0* ***SSE1::KanMX6*** | ***sse1∆*** | S288C | S288C/ EUROSCARF |
| **3** | ***sse2∆*** | MATa *his3Δ1 leu2Δ0 met15Δ0 ura3Δ0* ***SSE2::KanMX6*** | ***sse2∆*** | S288C | S288C/ EUROSCARF |
| **4** | ***yMPJ001*** | MATa *his3Δ1 leu2Δ0 met15Δ0 ura3Δ0* ***+ pRS315*** | **WT + EV** | S288C | This Study |
| **5** | ***yMPJ002*** | MATa *his3Δ1 leu2Δ0 met15Δ0 ura3Δ0 SSE1::KanMX6* ***+ pRS315*** | ***sse1∆* + EV** | S288C | This Study |
| **6** | ***yMPJ003*** | MATa *his3Δ1 leu2Δ0 met15Δ0 ura3Δ0 SSE1::KanMX6* ***+ pJV340*** | ***sse1∆* + EV-OE** | S288C | This Study |
| **7** | ***yMPJ004*** | MATa *his3Δ1 leu2Δ0 met15Δ0 ura3Δ0 SSE1::KanMX6* ***+ pRS315-SSE1*** | ***sse1∆* + SSE1** | S288C | This Study |
| **8** | ***yMPJ005*** | MATa *his3Δ1 leu2Δ0 met15Δ0 ura3Δ0 SSE1::KanMX6* ***+ pJV340-SSE1*** | ***sse1∆* + SSE1-OE** | S288C | This Study |
| **9** | ***yMPJ006*** | MATa *his3Δ1 leu2Δ0 met15Δ0 ura3Δ0 SSE1::KanMX6* ***+ pJV340-FES1*** | ***sse1∆* + FES1-OE** | S288C | This Study |
| **10** | ***yMPJ007*** | MATa *his3Δ1 leu2Δ0 met15Δ0 ura3Δ0 SSE1::KanMX6* ***+ pRS315-SSE1-K69Q*** | ***sse1∆* + SSE1-K69Q** | S288C | This Study |
| **11** | ***yMPJ008*** | MATa *his3Δ1 leu2Δ0 met15Δ0 ura3Δ0 SSE1::KanMX6* ***+ pRS315-SSE1-G205D*** | ***sse1∆* + SSE1-G205D** | S288C | This Study |
| **12** | ***yMPJ009*** | MATa *his3Δ1 leu2Δ0 met15Δ0 ura3Δ0 SSE1::KanMX6* ***+ pRS315-SSE1-G233D*** | ***sse1∆* + SSE1-G233D** | S288C | This Study |
| **13** | ***yMPJ010*** | MATa *his3Δ1 leu2Δ0 met15Δ0 ura3Δ0* ***+ pRS316*** | **WT + EV** | S288C | This Study |
| **14** | ***yMPJ011*** | MATa *his3Δ1 leu2Δ0 met15Δ0 ura3Δ0* ***+ BG1805-SSE2*** | **WT + SSE2-OE** | S288C | This Study |
| **15** | ***yMPJ012*** | MATa *his3Δ1 leu2Δ0 met15Δ0 ura3Δ0 SSE1::KanMX6* ***+ pRS316*** | ***sse1∆* + EV** | S288C | This Study |
| **16** | ***yMPJ013*** | MATa *his3Δ1 leu2Δ0 met15Δ0 ura3Δ0 SSE1::KanMX6* ***+ BG1805-SSE2*** | ***sse1∆* + SSE2-OE** | S288C | This Study |
| **17** | ***yMPJ014*** | MATa *his3Δ1 leu2Δ0 met15Δ0 ura3Δ0 SSE2::KanMX6* ***+ pRS316*** | ***sse2∆* + EV** | S288C | This Study |
| **18** | ***yMPJ015*** | MATa *his3Δ1 leu2Δ0 met15Δ0 ura3Δ0 SSE2::KanMX6* ***+ BG1805-SSE2*** | ***sse2∆* + SSE2-OE** | S288C | This Study |
| **19** | ***hac1∆*** | MATa *his3Δ1 leu2Δ0 met15Δ0 ura3Δ0* ***HAC1::KanMX6*** | ***hac1∆*** | S288C | S288C/ EUROSCARF |
| **20** | ***ire1∆*** | MATa *his3Δ1 leu2Δ0 met15Δ0 ura3Δ0* ***IRE1::KanMX6*** | ***ire1∆*** | S288C | S288C/ EUROSCARF |
| **21** | ***yMPJ016*** | MATa *his3Δ1 leu2Δ0 met15Δ0 ura3Δ0 HAC1::KanMX6* ***SSE1::His3MX6*** | ***hac1∆-sse1∆*** | S288C | This Study |
| **22** | ***yMPJ017*** | MATa *his3Δ1 leu2Δ0 met15Δ0 ura3Δ0 IRE1::KanMX6* ***SSE1::His3MX6*** | ***ire1∆-sse1∆*** | S288C | This Study |
| **23** | ***ssa1∆*** | MATa *his3Δ1 leu2Δ0 met15Δ0 ura3Δ0* ***SSA1::KanMX6*** | ***ssa1∆*** | S288C | S288C/ EUROSCARF |
| **24** | ***ssa2∆*** | MATa *his3Δ1 leu2Δ0 met15Δ0 ura3Δ0* ***SSA2::KanMX6*** | ***ssa2∆*** | S288C | S288C/ EUROSCARF |
| **25** | ***get1∆*** | MATa *his3Δ1 leu2Δ0 met15Δ0 ura3Δ0* ***GET1::KanMX6*** | ***get1∆*** | S288C | S288C/ EUROSCARF |
| **26** | ***ino2∆*** | MATa *his3Δ1 leu2Δ0 met15Δ0 ura3Δ0* ***INO2::KanMX6*** | ***ino2∆*** | S288C | S288C/ EUROSCARF |
| **27** | ***ino4∆*** | MATa *his3Δ1 leu2Δ0 met15Δ0 ura3Δ0* ***INO4::KanMX6*** | ***ino4∆*** | S288C | S288C/ EUROSCARF |
| **28** | ***ssb1∆*** | MATa *his3Δ1 leu2Δ0 met15Δ0 ura3Δ0* ***SSB1::KanMX6*** | ***ssb1∆*** | S288C | S288C/ EUROSCARF |
| **29** | ***yMPJ018*** | MATa *his3Δ1 leu2Δ0 met15Δ0 ura3Δ0* ***+ pRS316-HSF1*** | **WT + HSF1** | S288C | This Study |
| **30** | ***yMPJ019*** | MATa *his3Δ1 leu2Δ0 met15Δ0 ura3Δ0* ***+ pRS316-HSF1-R206S*** | **WT + HSF1-R206S** | S288C | This Study |
| **31** | ***yMPJ020*** | MATa *his3Δ1 leu2Δ0 met15Δ0 ura3Δ0 SSE1::KanMX6* ***+ pRS316-HSF1*** | ***sse1∆* + HSF1** | S288C | This Study |
| **32** | ***yMPJ021*** | MATa *his3Δ1 leu2Δ0 met15Δ0 ura3Δ0 SSE1::KanMX6* ***+ pRS316-HSF1-R206S*** | ***sse1∆* + HSF1-R206S** | S288C | This Study |
| **33** | ***ssz1∆*** | MATa *his3Δ1 leu2Δ0 met15Δ0 ura3Δ0* ***SSZ1::KanMX6*** | ***ssz1∆*** | S288C | S288C/ EUROSCARF |
| **34** | ***zuo1∆*** | MATa *his3Δ1 leu2Δ0 met15Δ0 ura3Δ0* ***ZUO1::KanMX6*** | ***zuo1∆*** | S288C | S288C/ EUROSCARF |
| **35** | ***jjj1∆*** | MATa *his3Δ1 leu2Δ0 met15Δ0 ura3Δ0* ***JJJ1::KanMX6*** | ***jjj1∆*** | S288C | S288C/ EUROSCARF |
| **36** | ***snl1∆*** | MATa *his3Δ1 leu2Δ0 met15Δ0 ura3Δ0* ***SNL1::KanMX6*** | ***snl1∆*** | S288C | S288C/ EUROSCARF |
| **37** | ***gim2∆*** | MATa *his3Δ1 leu2Δ0 met15Δ0 ura3Δ0* ***GIM2::KanMX6*** | ***gim2∆*** | S288C | S288C/ EUROSCARF |
| **38** | ***gim3∆*** | MATa *his3Δ1 leu2Δ0 met15Δ0 ura3Δ0* ***GIM3::KanMX6*** | ***gim3∆*** | S288C | S288C/ EUROSCARF |
| **39** | ***gim5∆*** | MATa *his3Δ1 leu2Δ0 met15Δ0 ura3Δ0* ***GIM5::KanMX6*** | ***gim5∆*** | S288C | S288C/ EUROSCARF |
| **40** | ***cpr6∆*** | MATa *his3Δ1 leu2Δ0 met15Δ0 ura3Δ0* ***CPR6::KanMX6*** | ***cpr6∆*** | S288C | S288C/ EUROSCARF |
| **41** | ***cct8∆*** | MATa *his3Δ1 leu2Δ0 met15Δ0 ura3Δ0* ***CCT8::KanMX6*** | ***cct8∆*** | S288C | S288C/ EUROSCARF |
| **42** | ***egd1∆*** | MATa *his3Δ1 leu2Δ0 met15Δ0 ura3Δ0* ***EGD1::KanMX6*** | ***egd1∆*** | S288C | S288C/ EUROSCARF |
| **43** | ***yMPJ022*** | MATa *his3Δ1 leu2Δ0 met15Δ0 ura3Δ0 JJJ1::KanMX6* ***SSE1::His3MX6*** | ***jjj1∆-sse1∆*** | S288C | This Study |
| **44** | ***yMPJ023*** | MATa *his3Δ1 leu2Δ0 met15Δ0 ura3Δ0 GIM2::KanMX6* ***SSE1::His3MX6*** | ***gim2∆-sse1∆*** | S288C | This Study |
| **45** | ***yMPJ024*** | MATa *his3Δ1 leu2Δ0 met15Δ0 ura3Δ0 CCT8::KanMX6* ***SSE1::His3MX6*** | ***cct8∆-sse1∆*** | S288C | This Study |
| **46** | ***yMJ003*** | MATα *his3Δ1 leu2Δ0 met15Δ0 ura3Δ0 LYS+Δcan1::STE2pr-spHIS5 Δlyp1::STE3pr-LEU2 cyh2 Δura3::****UPRE-GFP-TEF2pr-RFP-MET15-URA3*** | **UPR reporter strain** | S288C | Jonikas et al., 2009 |
| **47** | ***yVC002*** | MATα *his3Δ1 leu2Δ0 met15Δ0 ura3Δ0 LYS+Δcan1::STE2pr-spHIS5 Δlyp1::STE3pr-LEU2 cyh2 Δura3::UPRE-GFP-TEF2pr-RFP-MET15-URA3* ***SSE1::KanMX6*** | ***sse1∆* with UPR reporter** | S288C | This Study |
| **48** | ***yVC004*** | MATα *his3Δ1 leu2Δ0 met15Δ0 ura3Δ0 LYS+Δcan1::STE2pr-spHIS5 Δlyp1::STE3pr-LEU2 cyh2 Δura3::UPRE-GFP-TEF2pr-RFP-MET15-URA3* ***SSE2::KanMX6*** | ***sse2∆* with UPR reporter** | S288C | This Study |
| **49** | ***Pdi1_GFP_*** | MATa *his3Δ1 leu2Δ0 met15Δ0 ura3Δ0* ***PDI1-GFP- His3MX6*** | ***Pdi1* with GFP tag** | S288C | S288C/ Thermo |
| **50** | ***Lhs1_GFP_*** | MATa *his3Δ1 leu2Δ0 met15Δ0 ura3Δ0* ***LHS1-GFP::His3MX6*** | ***Lhs1* with GFP tag** | S288C | S288C/ Thermo |
| **51** | ***Sec62_GFP_*** | MATa *his3Δ1 leu2Δ0 met15Δ0 ura3Δ0* ***Sec62-GFP-His3MX6-GFP*** | ***Sec62* with GFP tag** | S288C | S288C/ Thermo |
| **52** | ***Ubc7_GFP_*** | MATa *his3Δ1 leu2Δ0 met15Δ0 ura3Δ0* ***UBC7-GFP-His3MX6*** | ***Ubc7* with GFP tag** | S288C | S288C/ Thermo |
| **53** | ***yMPJ025*** | MATa *his3Δ1 leu2Δ0 met15Δ0 ura3Δ0 PDI1::His3MX6-GFP* ***SSE1::URA3*** | ***Pdi1_GFP_-sse1∆*** | S288C | This Study |
| **54** | ***yMPJ026*** | MATa *his3Δ1 leu2Δ0 met15Δ0 ura3Δ0 LHS1::His3MX6-GFP* ***SSE1::URA3*** | ***Lhs1_GFP_-sse1∆*** | S288C | This Study |
| **55** | ***yMPJ027*** | MATa *his3Δ1 leu2Δ0 met15Δ0 ura3Δ0 SEC62::His3MX6-GFP* ***SSE1::URA3*** | ***Sec62_GFP_-sse1∆*** | S288C | This Study |
| **56** | ***yMPJ028*** | MATa *his3Δ1 leu2Δ0 met15Δ0 ura3Δ0 UBC7::His3MX6-GFP* ***SSE1::URA3*** | ***Ubc7_GFP_-sse1∆*** | S288C | This Study |
