# Supplementary figures and images for: Sse1, Hsp110 chaperone of yeast, controls the cellular fate during endoplasmic reticulum stress
Source: G3 (Bethesda). 2024 Apr 5;14(6):jkae075. doi: 10.1093/g3journal/jkae075 (PMC11152076; doi:10.1093/g3journal/jkae075)

Figure S1

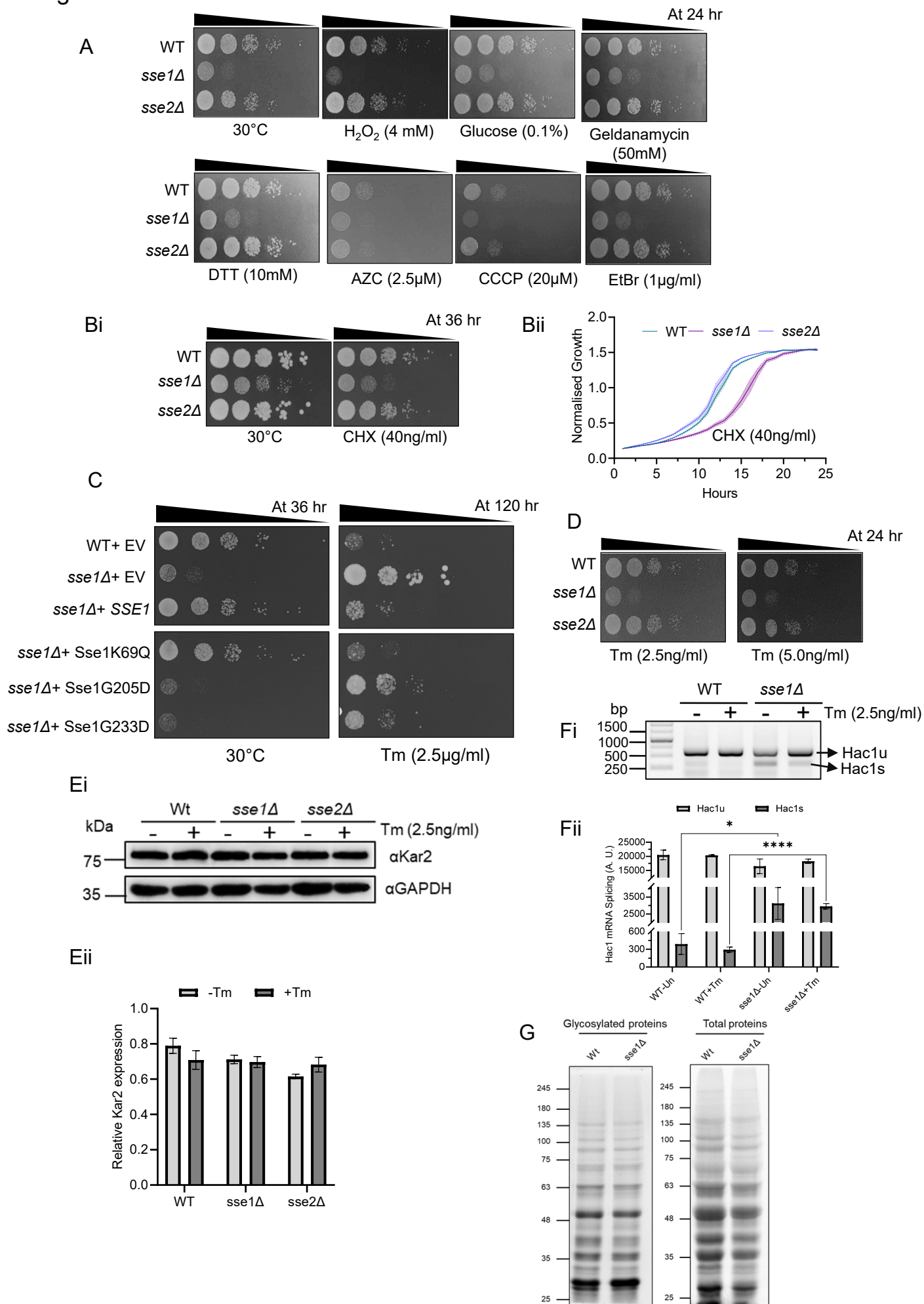

Supplement: jkae075_Supplementary_Data [file jkae075_supplementary_data.zip › Figure_S1_G3-2024-404855.pdf]

Figure S2

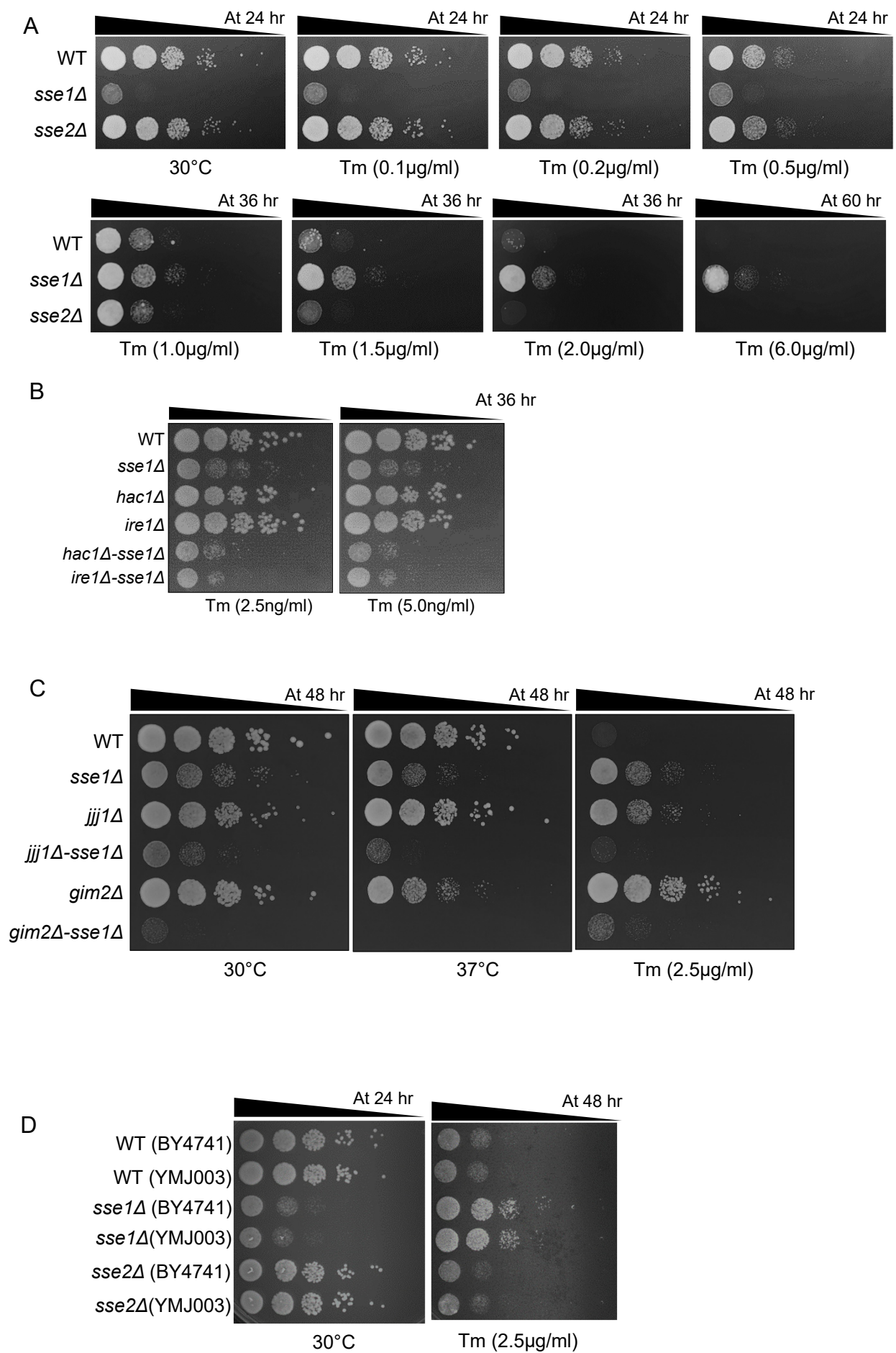

Supplement: jkae075_Supplementary_Data [file jkae075_supplementary_data.zip › Figure_S2_G3-2024-404855.pdf]

Figure S3

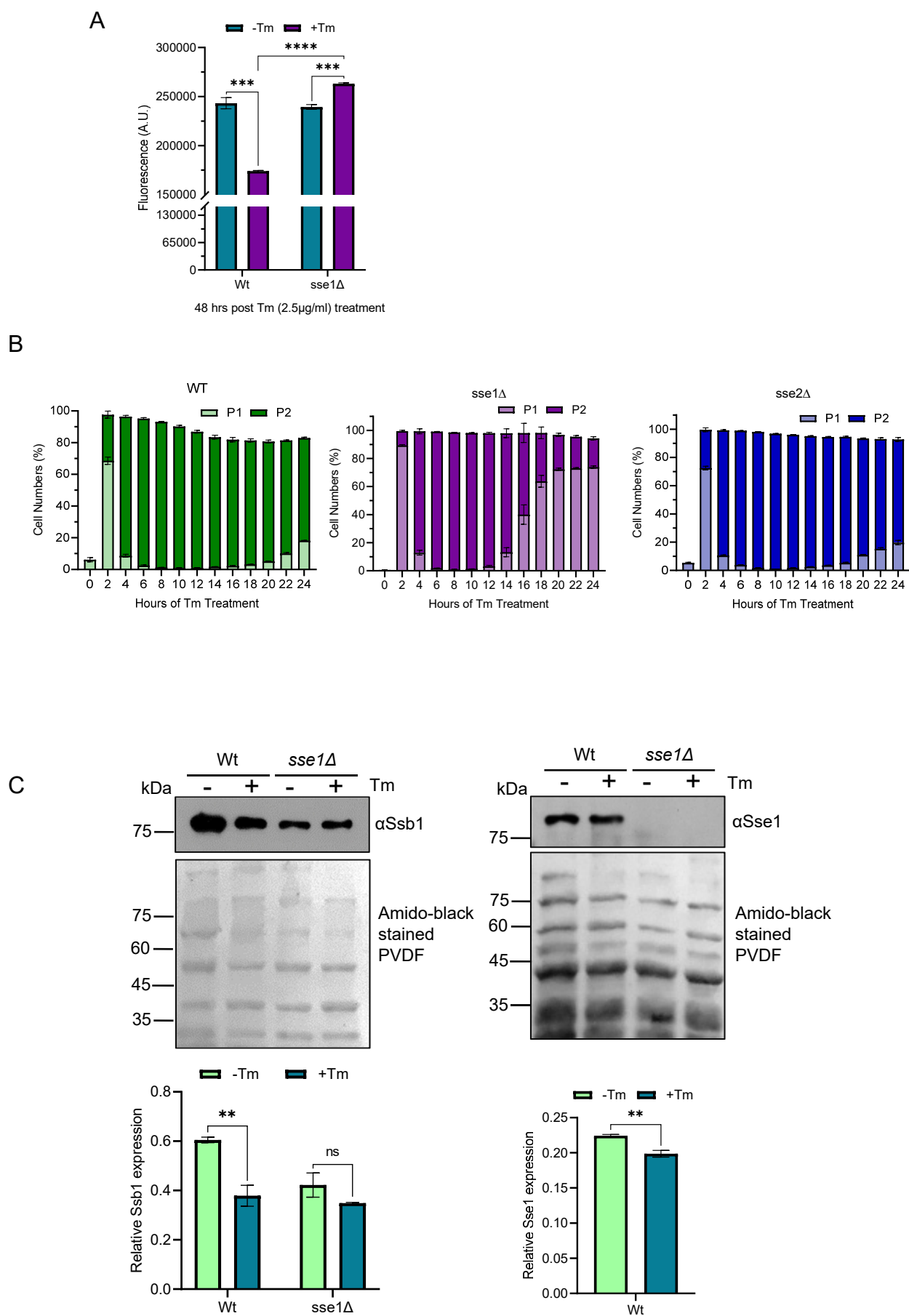

Supplement: jkae075_Supplementary_Data [file jkae075_supplementary_data.zip › Figure_S3_G3-2024-404855.pdf]

Figure S4

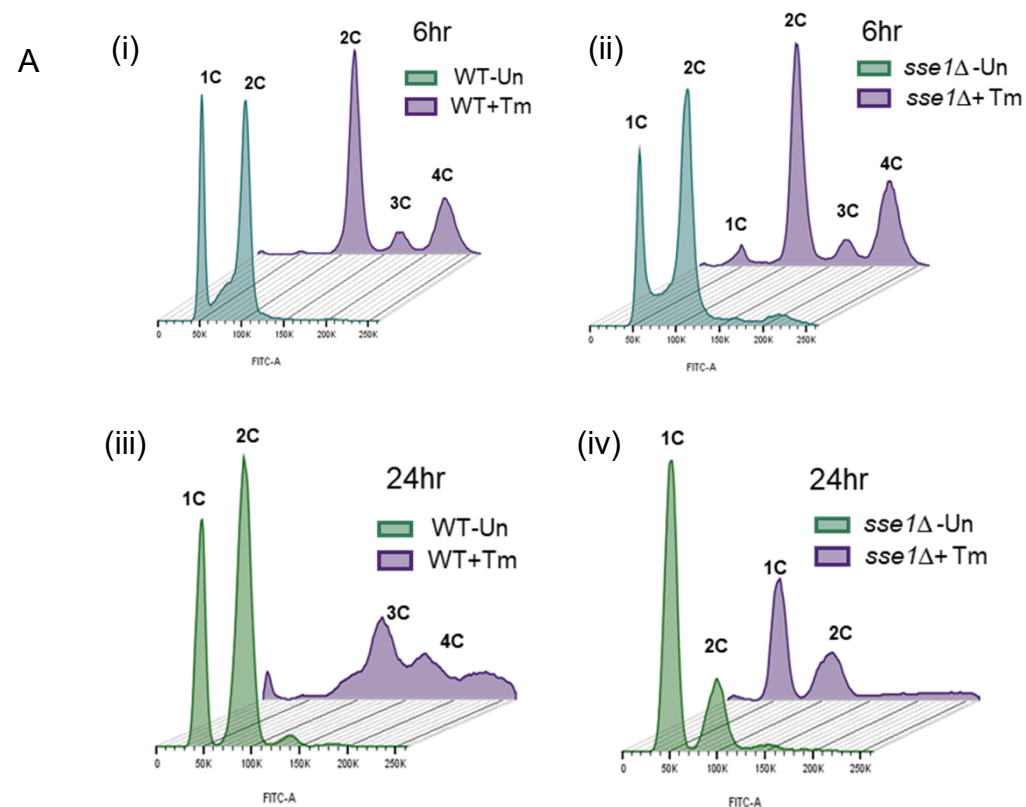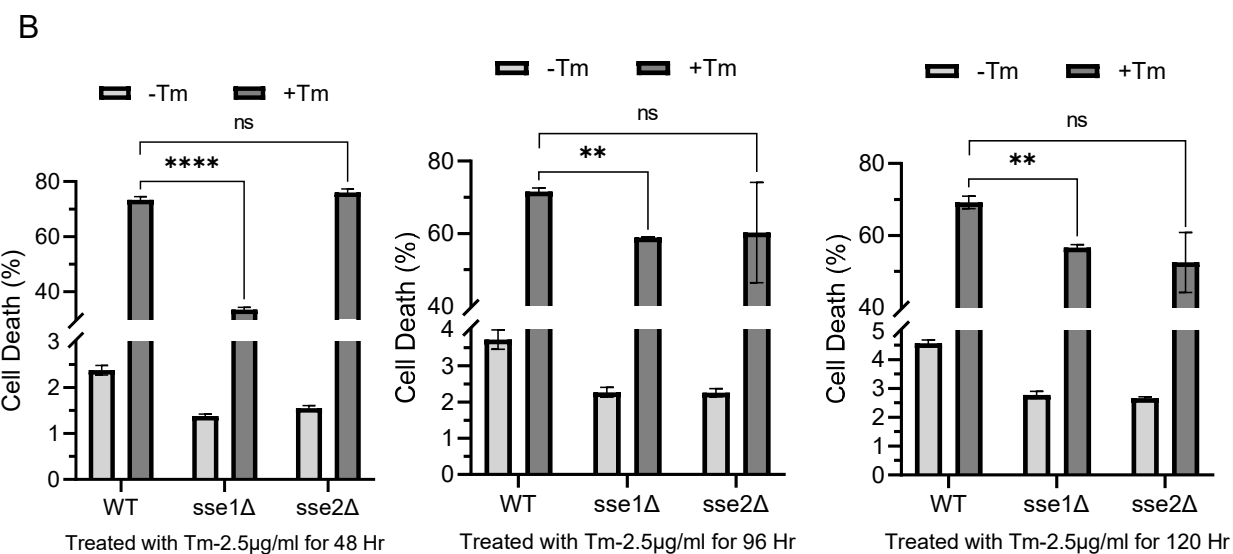

Supplement: jkae075_Supplementary_Data [file jkae075_supplementary_data.zip › Figure_S4_G3-2024-404855.pdf]

Figure S5

A

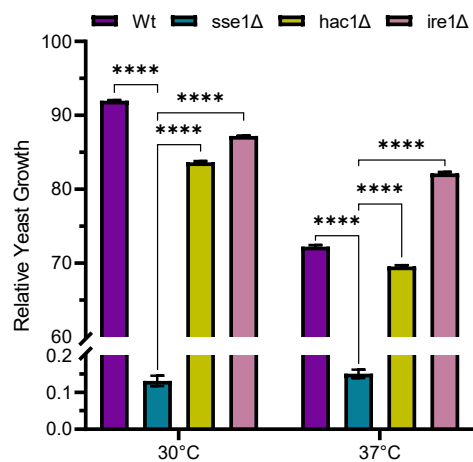

**B**

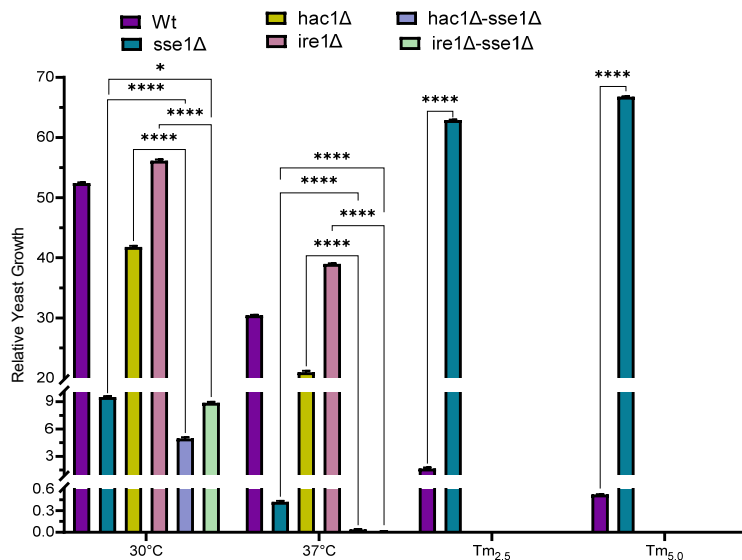

C

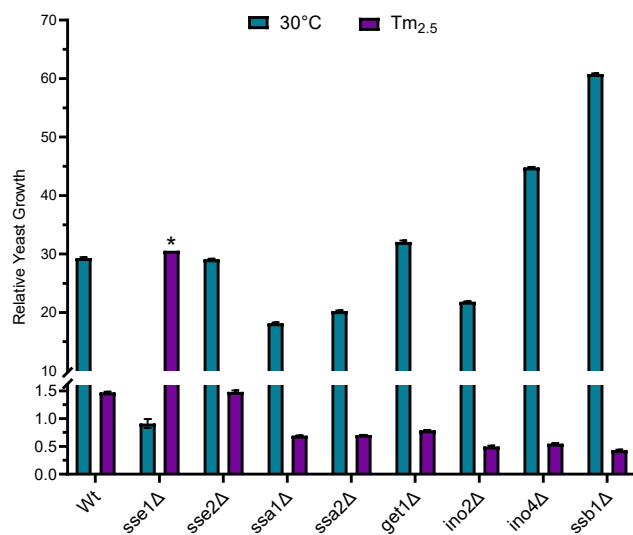

D

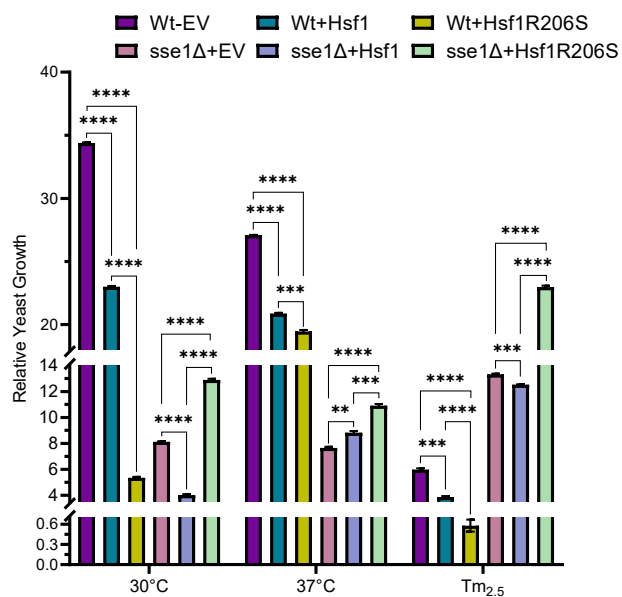

E

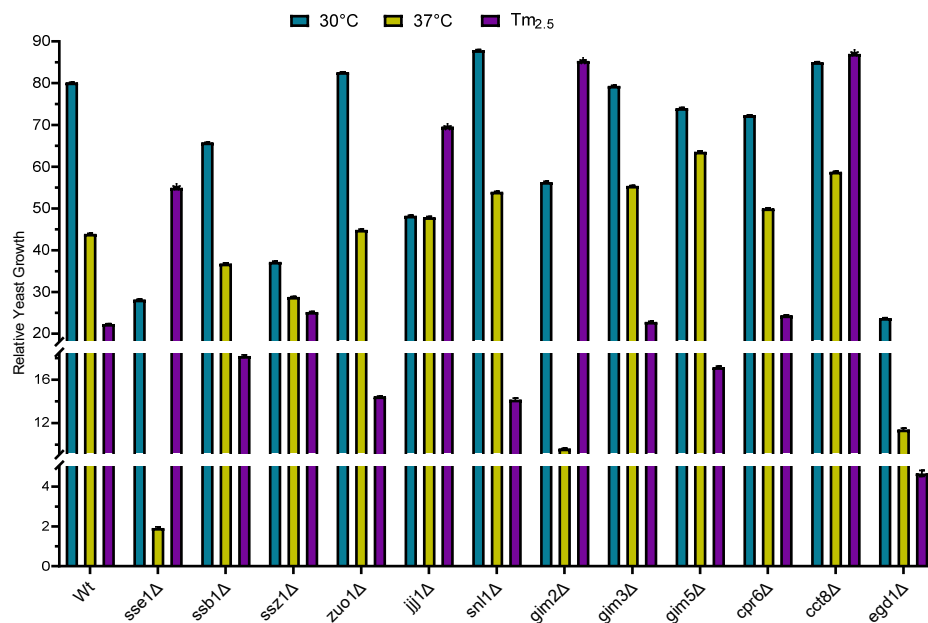

Supplement: jkae075_Supplementary_Data [file jkae075_supplementary_data.zip › Figure_S5_G3-2024-404855.pdf]
